# Robotic vs. laparoscopic inguinal hernia repair in patients with chronic kidney disease: a propensity score-matched analysis

**DOI:** 10.1007/s11701-026-03782-y

**Published:** 2026-08-18

**Authors:** Fouad Hanna, Doaa Bayomi, Sara T. Zaki, Ali Dway, Mohamed H. Zidan, Mahmoud Albashier, Ahmed Amgad

**Affiliations:** 1 Faculty of Medicine, Cairo University, Giza, Egypt; 2 Faculty of Medicine, Alexandria University, Alexandria, Egypt; 3 Faculty of Pharmacy, Badr University, Cairo, Egypt; 4 Al-Andalus University for Medical Sciences, Tartus, Syria; 5 Faculty of Medicine, Damietta University, New Damietta, Egypt; 6 Faculty of Medicine, Capital University (formerly Helwan University), Cairo, Egypt; 7 Surgical Undergraduate Training & Unified Research Education (SUTURE), Cairo, Egypt

**Keywords:** Hernia repair, Chronic kidney disease, Robotic-assisted surgery, Laparoscopic surgery, Acute kidney injury, Propensity score matching

## Abstract

CKD patients are susceptible to pneumoperitoneum-induced renal injury; comparative outcomes of robotic versus laparoscopic inguinal hernia repair are unknown. Retrospective propensity score–matched cohort using TriNetX (184 HCOs; 2015–2026). CKD adults undergoing robotic or laparoscopic inguinal hernia repair were matched 1:1 on 61 covariates (1,693 per arm at 1 month; 1,659 at 6 months). Primary outcomes were AKI and MACE; secondary outcomes were metabolic acidosis, urinary retention, hyperkalemia, and post-operative eGFR and creatinine. Cox regression, Kaplan–Meier, and competing-risk CIF were used, with E-values for unmeasured confounding. At 1 month, robotic repair had higher AKI (5.0% vs. 2.9%; HR 1.74, 95% CI 1.22–2.47; *p* = 0.002) and MACE (2.5% vs. 1.4%; HR 1.84; *p* = 0.017). At 6 months, AKI persisted (HR 1.41; *p* = 0.002) and urinary retention emerged (HR 1.33; *p* = 0.039); MACE (*p* = 0.112) and acidosis (*p* = 0.199) did not. New-onset hyperkalemia was lower with robotic (HR 0.58; *p* = 0.045). eGFR MDRD declined less at 1 month (Δ − 1.28 vs. − 3.21; *p* = 0.049); 6-month creatinine was lower (*p* = 0.013). CIF (deaths 1.5% vs. 1.9%) confirmed AKI; KM overstated 6-month incidence by 1.1–1.9 points. 1-month AKI E-value 2.87 (CI 1.74). Robotic inguinal hernia repair in CKD was associated with higher AKI and early MACE in the all-events analysis, but the incident AKI signal was borderline and post-operative laboratory renal function favored the robotic arm. This discordance may reflect differential ascertainment at robotic-predominant academic centers. These hypothesis-generating findings do not establish a renal safety advantage for either approach; prospective validation with KDIGO-defined AKI endpoints is required.

**Graphical abstract:**

**Figure:**
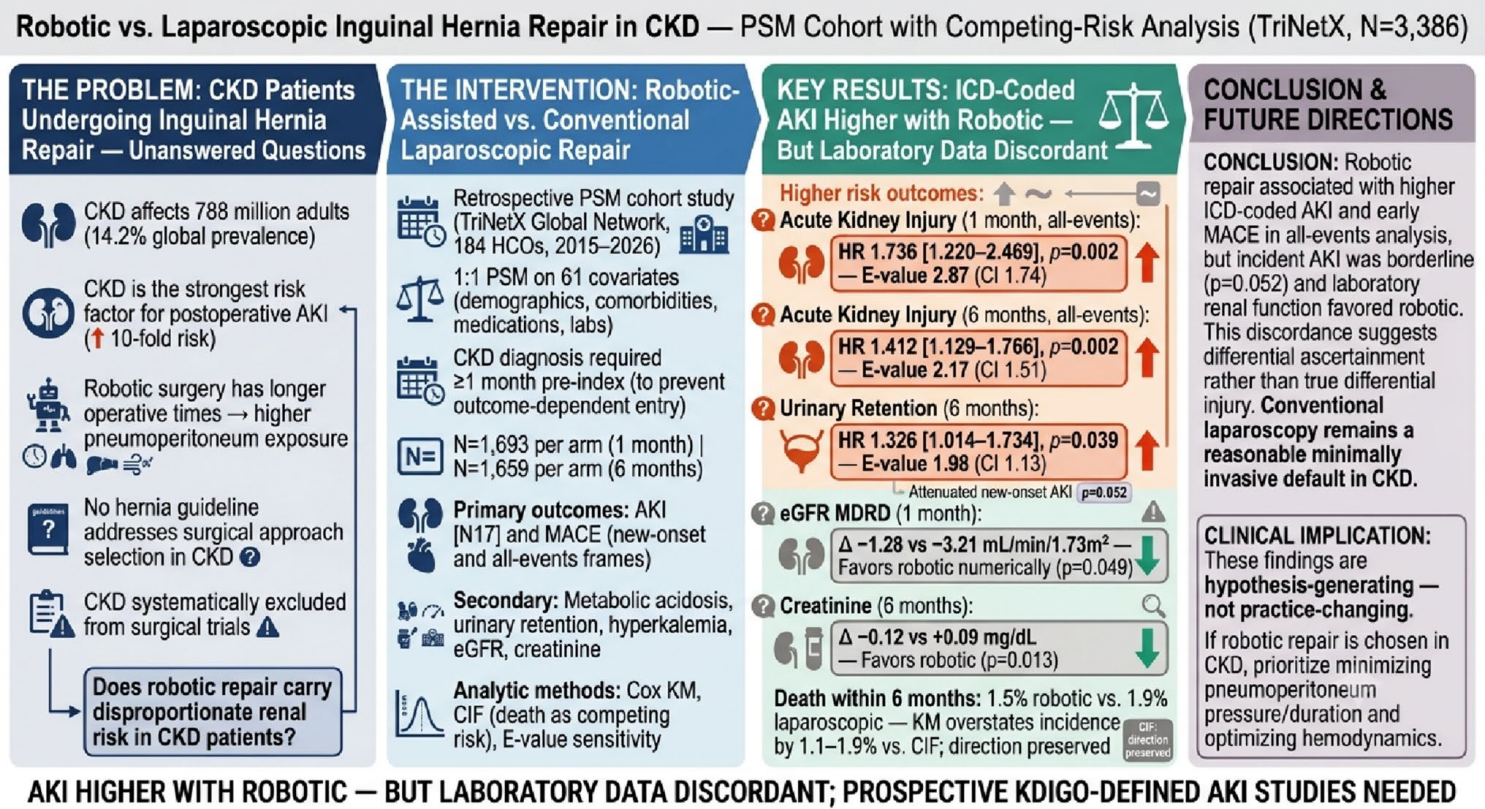

**Supplementary Information:**

The online version contains supplementary material available at 10.1007/s11701-026-03782-y.

## Introduction

Inguinal hernia repair is among the most common operations worldwide, with an estimated 20 million procedures annually and a lifetime risk of 27% in men and 3% in women [1–3]. The Global Burden of Disease Study 2021 reported 13 million incident cases in 2019, with the greatest burden in males, older adults, and low- to middle-income countries [1, 4]. Minimally invasive techniques offer reduced postoperative pain, earlier recovery, and recurrence comparable to open repair [5], and robotic-assisted repair has grown rapidly from 0.3% to 2.6% of groin hernia repairs among Medicare beneficiaries between 2010 and 2021 [6]. 

Despite this growth, virtually all comparative studies of robotic versus laparoscopic inguinal hernia repair have been conducted in general surgical populations without stratification by renal function. This is consequential given the global burden of chronic kidney disease (CKD), which affects an estimated 788 million adults (14.2%) and is the ninth leading cause of death worldwide [7]. CKD is the single strongest risk factor for postoperative acute kidney injury (AKI), conferring up to a 10-fold increase in risk [8]. Yet no hernia guideline including the HerniaSurge and European Hernia Society recommendations addresses surgical-approach selection or the differential renal risks of robotic versus laparoscopic techniques in CKD [2, 9]. 

No study has examined whether the choice between robotic and laparoscopic approaches differentially affects renal and cardiovascular outcomes in this high-risk population. Using the TriNetX Global Collaborative Network, we therefore conducted a propensity score-matched retrospective cohort study comparing postoperative renal and cardiovascular outcomes between robotic-assisted and conventional laparoscopic inguinal hernia repair in patients with pre-existing CKD.

## Materials and methods

### Study Design

This retrospective propensity score–matched cohort study used de-identified aggregate data from the TriNetX Analytics Network. All data are certified as fully de-identified under HIPAA Sect. 164.514(a); institutional review board approval and individual informed consent were therefore waived. The study followed the Declaration of Helsinki and is reported in accordance with STROBE (checklist in Supplementary Table S-STROBE) [10]. Subgroup analyses and outcome definitions were pre-specified internally by the study team before data extraction; no external protocol registration was performed.

### Data Source

Data were drawn from the TriNetX Global Collaborative Network, a federated platform aggregating anonymized electronic health record data from participating healthcare organizations (HCOs). At analysis (July 2026) the network comprised 184 HCOs, predominantly in the United States, spanning academic and community systems [11]. Diagnoses are coded with ICD-10-CM; procedures with CPT and ICD-10-PCS; laboratory results with LOINC; and medications with RxNorm. Robotic cases were contributed by 49 of the 184 queried HCOs and laparoscopic cases by 81; this differential representation is addressed in the Limitations.

### Study Population and Cohort Definitions

**Fig. 1:**
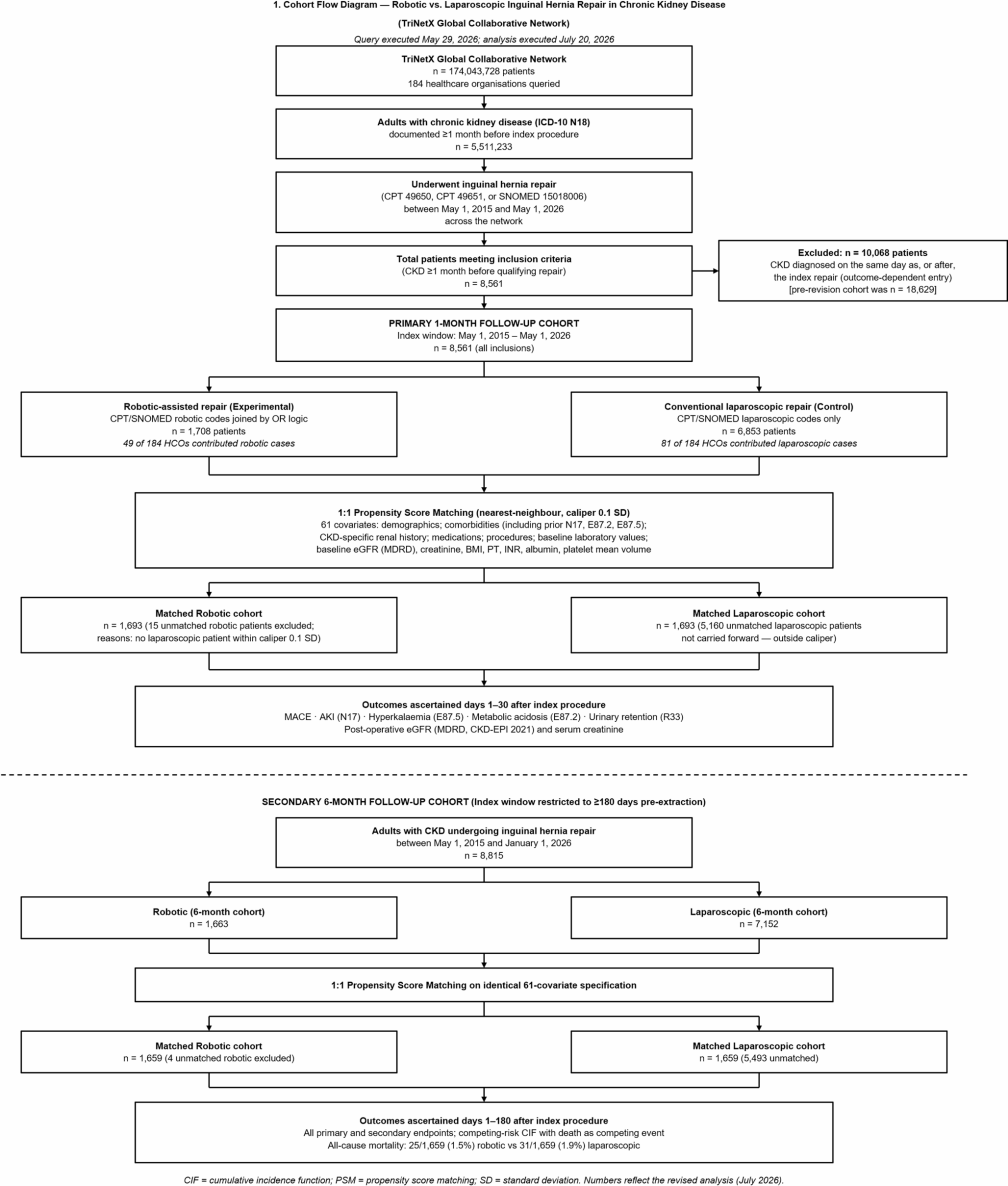
Cohort Flow Diagram

Adults with CKD (ICD-10 N18) who underwent robotic-assisted (Cohort 1) or conventional laparoscopic (Cohort 2) inguinal hernia repair were identified. To prevent outcome-dependent cohort entry whereby a patient who develops post-operative AKI could subsequently acquire a CKD code and enter the study CKD was required to be documented at least 1 month before the index procedure. This restriction reduced the eligible population from 18,629 to 8,561 patients (1,708 robotic; 6,853 laparoscopic), a 54% reduction (Fig. 1).

### Selection of the chronic kidney disease code set

The primary research question addressed by this study is general rather than stage-specific: whether the choice between robotic-assisted and conventional laparoscopic inguinal hernia repair is associated with different renal and cardiovascular outcomes among patients who have chronic kidney disease of any severity. Cohort entry was therefore defined by the parent ICD-10-CM category N18 (chronic kidney disease), which captures the entire hierarchy of child codes and yields the complete, unselected population of CKD patients undergoing minimally invasive inguinal hernia repair across the network. Restricting cohort entry to the stage-specified subcodes alone would have introduced a systematic selection bias, because the availability of a stage-specified code reflects the completeness of a patient’s renal work-up and the documentation practices of the treating institution, rather than the presence or severity of kidney disease itself.

Within the N18 hierarchy it is necessary to distinguish two categorically different types of code. The stage-specified codes N18.1 (stage 1), N18.2 (stage 2, mild), N18.3 (stage 3, moderate), N18.4 (stage 4, severe), N18.5 (stage 5), and N18.6 (end-stage renal disease) assign each patient to a defined KDIGO severity stratum and carry explicit prognostic information about the degree of renal impairment. The unspecified code, N18.9 (chronic kidney disease, unspecified), records the presence of chronic kidney disease without assigning a stage. A patient carrying N18.9 has documented CKD, but the coded record does not indicate whether that disease is mild or advanced. N18.9 is therefore informative for cohort membership but uninformative for severity stratification.

Because the parent N18 code encompasses both types, our main cohort necessarily includes both stage-specified and unspecified patients. In the matched 1-month cohort, 1,079 robotic and 1,076 laparoscopic patients carried a stage-specified code, while 614 and 617 respectively carried only N18.9 (Table 1). Retaining the unspecified group in the main analysis preserves the representativeness and statistical power of the primary comparison; excluding it from the stage-stratified subgroup analyses preserves the interpretability of those analyses.

**Table 1:** Baseline Characteristics Before and After Propensity Score Matching

Characteristic	Before Matching			After Matching		
	Robotic (*N* = 1,708)	Laparoscopic (*N* = 6,853)	SMD	Robotic (*N* = 1,693)	Laparoscopic (*N* = 1,693)	SMD
*Demographics*						
Age, years (mean ± SD)	69.9 ± 11.6	69.6 ± 13.4	0.021	69.9 ± 11.7	70.4 ± 11.9	0.043
Male, n (%)	1,529 (89.5%)	6,227 (90.9%)	0.045	1,516 (89.5%)	1,518 (89.7%)	0.004
White, n (%)	1,299 (76.1%)	5,084 (74.2%)	0.043	1,287 (76.0%)	1,323 (78.1%)	0.051
Black or African American, n (%)	251 (14.7%)	990 (14.4%)	0.007	249 (14.7%)	218 (12.9%)	0.053
Not Hispanic or Latino, n (%)	1,486 (87.0%)	5,136 (74.9%)	0.311	1,471 (86.9%)	1,470 (86.8%)	0.002
*Comorbidities*						
Essential hypertension, n (%)	1,073 (62.8%)	4,345 (63.4%)	0.012	1,062 (62.7%)	1,042 (61.5%)	0.024
Heart failure, n (%)	270 (15.8%)	973 (14.2%)	0.045	266 (15.7%)	258 (15.2%)	0.013
Cardiomegaly, n (%)	98 (5.7%)	283 (4.1%)	0.074	94 (5.6%)	102 (6.0%)	0.020
Type 2 diabetes mellitus, n (%)	504 (29.5%)	1,812 (26.4%)	0.068	495 (29.2%)	494 (29.2%)	0.001
Disorders of lipoprotein metabolism, n (%)	946 (55.4%)	3,850 (56.2%)	0.016	935 (55.2%)	924 (54.6%)	0.013
Obesity, n (%)	75 (4.4%)	223 (3.3%)	0.059	71 (4.2%)	68 (4.0%)	0.009
COPD, n (%)	170 (10.0%)	538 (7.9%)	0.074	162 (9.6%)	160 (9.5%)	0.004
Emphysema, n (%)	62 (3.6%)	179 (2.6%)	0.059	58 (3.4%)	56 (3.3%)	0.007
Pneumonia, n (%)	42 (2.5%)	150 (2.2%)	0.018	41 (2.4%)	44 (2.6%)	0.011
Asthma, n (%)	90 (5.3%)	356 (5.2%)	0.003	89 (5.3%)	99 (5.8%)	0.026
Pulmonary collapse, n (%)	47 (2.8%)	135 (2.0%)	0.052	45 (2.7%)	43 (2.5%)	0.007
Pulmonary embolism, n (%)	19 (1.1%)	68 (1.0%)	0.012	19 (1.1%)	20 (1.2%)	0.006
DVT (acute), n (%)	25 (1.5%)	100 (1.5%)	< 0.001	24 (1.4%)	25 (1.5%)	0.005
DVT (chronic), n (%)	10 (0.6%)	30 (0.4%)	0.021	10 (0.6%)	10 (0.6%)	< 0.001
*Cardiovascular History*						
Acute MI, n (%)	58 (3.4%)	158 (2.3%)	0.066	57 (3.4%)	62 (3.7%)	0.016
STEMI, n (%)	12 (0.7%)	29 (0.4%)	0.037	12 (0.7%)	10 (0.6%)	0.015
NSTEMI, n (%)	26 (1.5%)	75 (1.1%)	0.038	26 (1.5%)	32 (1.9%)	0.027
Old MI, n (%)	120 (7.0%)	382 (5.6%)	0.060	117 (6.9%)	110 (6.5%)	0.017
Cerebral infarction, n (%)	44 (2.6%)	196 (2.9%)	0.017	43 (2.5%)	37 (2.2%)	0.023
Intracerebral hemorrhage, n (%)	10 (0.6%)	17 (0.2%)	0.052	10 (0.6%)	10 (0.6%)	< 0.001
Right heart failure, n (%)	10 (0.6%)	14 (0.2%)	0.061	10 (0.6%)	10 (0.6%)	< 0.001
Malignant neoplasm, n (%)	10 (0.6%)	28 (0.4%)	0.025	10 (0.6%)	10 (0.6%)	< 0.001
*CKD-Related Matching Variables*						
Prior AKI (N17), n (%)	192 (11.2%)	567 (8.3%)	0.100	184 (10.9%)	186 (11.0%)	0.004
Prior Acidosis (E87.2), n (%)	63 (3.7%)	218 (3.2%)	0.028	60 (3.5%)	61 (3.6%)	0.003
Prior Hyperkalemia (E87.5), n (%)	100 (5.9%)	315 (4.6%)	0.057	98 (5.8%)	109 (6.4%)	0.027
Creatinine, mg/dL (mean ± SD)	1.9 ± 4.1	2.0 ± 5.6	0.028	1.9 ± 4.1	2.1 ± 6.3	0.042
eGFR MDRD (mean ± SD)	53.3 ± 21.9	53.4 ± 22.0	0.008	53.2 ± 21.8	52.0 ± 22.2	0.055
eGFR CKD-EPI 2021 (mean ± SD)	56.3 ± 19.1	55.1 ± 20.8	0.060	55.8 ± 18.4	53.0 ± 20.7	0.143
*CKD Stage Distribution*						
Stage 1 [N18.1], n (%)	18 (1.1%)	90 (1.3%)	0.013	18 (1.1%)	20 (1.2%)	0.011
Stage 2 [N18.2], n (%)	132 (7.7%)	648 (9.5%)	0.032	132 (7.8%)	131 (7.7%)	0.002
Stage 3 [N18.3], n (%)	641 (37.5%)	2,874 (41.9%)	0.016	637 (37.6%)	627 (37.0%)	0.012
Stage 4 [N18.4], n (%)	138 (8.1%)	519 (7.6%)	0.042	136 (8.0%)	138 (8.2%)	0.004
Stage 5 [N18.5], n (%)	42 (2.5%)	274 (4.0%)	0.067	42 (2.5%)	45 (2.7%)	0.011
ESRD [N18.6], n (%)	114 (6.7%)	653 (9.5%)	0.074	114 (6.7%)	115 (6.8%)	0.002
Unspecified CKD [N18.9], n (%)	623 (36.5%)	1,795 (26.2%)	—	614 (36.3%)	617 (36.4%)	—
Aggregate: stages 1–3, n (%)	791 (46.3%)	3,612 (52.7%)	—	787 (46.5%)	778 (46.0%)	—
Aggregate: stages 4–6, n (%)	294 (17.2%)	1,446 (21.1%)	—	292 (17.2%)	298 (17.6%)	—
*Procedures*						
Arterial procedures, n (%)	26 (1.5%)	71 (1.0%)	0.043	23 (1.4%)	27 (1.6%)	0.020
Pacemaker/ICD, n (%)	10 (0.6%)	60 (0.9%)	0.034	10 (0.6%)	11 (0.6%)	0.008
CABG, n (%)	10 (0.6%)	10 (0.1%)	0.073	10 (0.6%)	10 (0.6%)	< 0.001
Prior hernia repair, n (%)	26 (1.5%)	61 (0.9%)	0.058	22 (1.3%)	25 (1.5%)	0.015
Colon/rectal surgery, n (%)	74 (4.3%)	324 (4.7%)	0.019	73 (4.3%)	73 (4.3%)	< 0.001
*Medications*						
Atorvastatin, n (%)	322 (18.9%)	1,313 (19.2%)	0.008	317 (18.7%)	310 (18.3%)	0.011
Rosuvastatin, n (%)	124 (7.3%)	433 (6.3%)	0.037	121 (7.1%)	128 (7.6%)	0.016
Labetalol, n (%)	77 (4.5%)	304 (4.4%)	0.003	75 (4.4%)	78 (4.6%)	0.009
Metoprolol, n (%)	247 (14.5%)	990 (14.4%)	< 0.001	240 (14.2%)	250 (14.8%)	0.017
Hydralazine, n (%)	154 (9.0%)	520 (7.6%)	0.052	147 (8.7%)	149 (8.8%)	0.004
Lisinopril, n (%)	184 (10.8%)	710 (10.4%)	0.013	182 (10.8%)	170 (10.0%)	0.023
Aspirin, n (%)	228 (13.3%)	803 (11.7%)	0.049	223 (13.2%)	222 (13.1%)	0.002
Clopidogrel, n (%)	71 (4.2%)	264 (3.9%)	0.016	70 (4.1%)	65 (3.8%)	0.015
Thiazide diuretics, n (%)	139 (8.1%)	596 (8.7%)	0.020	138 (8.2%)	136 (8.0%)	0.004
Loop diuretics, n (%)	213 (12.5%)	809 (11.8%)	0.020	207 (12.2%)	192 (11.3%)	0.027
*Laboratory Values*						
BMI, kg/m² (mean ± SD)	27.3 ± 4.9	26.8 ± 4.6	0.091	27.2 ± 4.9	26.9 ± 4.9	0.060
Platelet mean volume, fL (mean ± SD)	9.5 ± 1.4	10.0 ± 1.2	0.359	9.5 ± 1.4	9.9 ± 1.3	0.282
Albumin, g/dL (mean ± SD)	4.0 ± 0.5	4.1 ± 0.5	0.179	4.0 ± 0.5	4.0 ± 0.5	0.126
HbA1c, % (mean ± SD)	6.2 ± 1.1	6.3 ± 1.1	0.052	6.2 ± 1.1	6.2 ± 1.0	0.010
Heart rate,/min (mean ± SD)	72.4 ± 13.6	71.7 ± 14.7	0.048	72.4 ± 13.6	71.3 ± 13.5	0.077
Body weight, lbs (mean ± SD)	183.2 ± 40.1	181.8 ± 36.4	0.037	183.1 ± 39.9	182.2 ± 38.1	0.022
Prothrombin time (PT) in Plasma or Blood (mean ± SD)	13.4 ± 4.4	13.8 ± 5.0	0.083	13.4 ± 4.4	13.8 ± 5.1	0.094
Oxygen saturation, % (mean ± SD)	95.3 ± 8.4	94.0 ± 12.8	0.123	95.4 ± 8.3	93.8 ± 13.5	0.148

CKD stage was derived from the ICD-10-CM subcodes N18.1–N18.6, with N18.9 denoting chronic kidney disease of unspecified stage. Percentages are calculated against the total cohort size in each column and sum to 100% across all seven strata. Every individual CKD stage was already well balanced between the two arms before propensity score matching, with all stage-specific standardised mean differences below the conventional 0.10 balance threshold and a maximum pre-matching SMD of 0.074 (ESRD, N18.6). The additional matching on CKD stage reported here was therefore not required to achieve balance; it was undertaken as a deliberate robustness step to reduce residual imbalance still further — the maximum stage-specific SMD fell from 0.074 to 0.012 after matching — so that the direction of the observed outcome differences could be attributed with confidence to surgical approach rather than to any residual difference in underlying renal severity. Patients with unspecified CKD (N18.9) are shown for completeness and were retained in the main analysis, but were excluded from the stage-stratified subgroup analyses (see Methods, Subgroup Analyses). SMD, standardised mean difference; ESRD, end-stage renal disease

Baseline demographic, comorbidity, laboratory, medication, and procedural characteristics of robotic-assisted and conventional laparoscopic cohorts before and after 1:1 propensity score matching on 61 pre-specified covariates. Values are n (%) for categorical variables and mean ± SD for continuous variables. Standardized mean differences (SMDs) below 0.10 indicate satisfactory covariate balance. Residual imbalance exceeding 0.10 in a limited number of laboratory summaries (platelet mean volume, oxygen saturation, eGFR CKD-EPI 2021, albumin) reflects small denominators for these labs within the matched cohort and is addressed in the Limitations section. Abbreviations: AKI, acute kidney injury; BMI, body mass index; CABG, coronary artery bypass grafting; CKD, chronic kidney disease; COPD, chronic obstructive pulmonary disease; DVT, deep vein thrombosis; eGFR, estimated glomerular filtration rate; ICD, implantable cardioverter-defibrillator; MDRD, Modification of Diet in Renal Disease; MI, myocardial infarction; NSTEMI, non-ST-elevation myocardial infarction; PSM, propensity score matching; SD, standard deviation; SMD, standardized mean difference; STEMI, ST-elevation myocardial infarction

Cohort 1 required a laparoscopic inguinal hernia repair (CPT 49650 or 49651, or SNOMED 15018006) within one day of a robotic-assist code (HCPCS S2900 or ICD-10-PCS 8E0W4CZ), or any of the robotic hernia SNOMED codes (1156322007, 1156323002, or 1156324008; joined by OR logic). Cohort 2 required a laparoscopic inguinal hernia repair without any co-occurring robotic-assist or robotic-hernia code. The index event was the first qualifying repair meeting each cohort definition. All patients required CKD documented ≥ 1 month before the index procedure and an index procedure between 1 May 2015 and 1 May 2026. To ensure ≥ 180 days of potential follow-up in the 6-month analysis, that index window was restricted to 1 May 2015–1 January 2026.

Outcomes were ascertained over two pre-specified landmarks: day 1–30 (1-month follow-up) and day 1–180 (6-month follow-up). Ascertainment began at day 1 because TriNetX assigns procedure and diagnosis codes to the same calendar date; including day 0 would risk capturing pre-existing diagnoses documented during the index encounter. AKI recognized intra-operatively or in the immediate recovery period on the day of surgery may therefore be undercaptured, and this is acknowledged as a limitation. For Kaplan–Meier and Cox analyses, patients were censored at the last recorded clinical fact; for CIF, all-cause death within the 180-day window was treated as a competing event.

### Outcomes

Primary outcomes were AKI (ICD-10 N17) and MACE (composite of I21, I22, I63, or I46). Secondary outcomes were metabolic acidosis (E87.2), urinary retention (R33), hyperkalemia (E87.5), post-operative eGFR (MDRD [LOINC/TNX 8001] and CKD-EPI 2021 [LOINC 98980-6]) and serum creatinine (TNX 9024), and all-cause mortality (TriNetX deceased demographic flag). MDRD was retained as the primary laboratory eGFR because it was the most widely available measure across the network; CKD-EPI 2021, recommended by KDIGO 2024 and the NKF-ASN Task Force, was reported in parallel where available but with substantially smaller denominators [11–14]. The N17 code does not correspond to KDIGO-defined AKI and captures a heterogeneous spectrum from transient creatinine elevations to severe injury requiring renal replacement therapy; this is central to the interpretation and is addressed in the Discussion.

Each clinical outcome was analyzed under two pre-specified analytic frames: (i) an all-events frame including patients with the outcome documented before the index date, reflecting total clinical burden; and (ii) a new-onset frame excluding patients with any prior documentation of the outcome, isolating incident post-operative events. The new-onset frame is the primary analytic frame for causal inference. Within TriNetX, event counts below 10 are suppressed for patient privacy; at 1 month, new-onset event counts for AKI, MACE, metabolic acidosis, and hyperkalemia fell below this threshold in at least one arm and are not reported. Full code lists appear in Supplementary File 1.

### Propensity Score Matching

1:1 propensity score matching used a greedy nearest-neighbor algorithm with a caliper of 0.1 pooled standard deviations of the logit of the propensity score [15]. The propensity score model incorporated 61 covariates spanning demographics; pre-existing diagnoses (including prior N17, E87.2, and E87.5, added in this revision); medications (statins, antihypertensives, antiplatelet agents, diuretics); prior surgical procedures; and baseline laboratory values (eGFR MDRD, eGFR CKD-EPI 2021, creatinine, BMI, HbA1c, lipid panel, heart rate, oxygen saturation, albumin, platelet mean volume, PT, INR, body weight). The full list appears in Table 1. Calendar year of surgery and site-level characteristics (academic vs. community) are not available as matchable covariates on the TriNetX platform; era-stratified (2015–2019 vs. 2020–2026) and academic-center subgroups partially address the resulting temporal and institutional confounding but do not fully resolve it.

### Handling of missing laboratory data

TriNetX handles laboratory covariates with missing values through a complete-case within-covariate approach: patients without a recorded value in the specified lookback window are excluded from the matching distance for that covariate but remain eligible for matching on all other covariates [16]. No imputation was performed; patient-level multiple imputation is infeasible because TriNetX releases only aggregate summary statistics. Availability of every baseline laboratory covariate is reported in Supplementary Table S-Missing. The five specialized covariates recorded in fewer than 10% of patients (eGFR CKD-EPI 2021, urine albumin/creatinine, cardiac troponin I, BNP, NT-proBNP) contribute to residual standardized mean differences (SMDs) that mechanically inflate when denominators are small; balance on the widely available covariates most relevant to CKD physiology (creatinine, eGFR MDRD, weight, BMI, heart rate, albumin) provides the substantive basis for exchangeability.

SMDs were the primary balance metric, with SMD < 0.10 indicating satisfactory balance per Austin [15, 17]. Consistent with recommendations against significance testing in matched samples, post-matching p-values are not reported. SMDs for all 61 covariates appear in Supplementary Table S-Balance, including PT and INR.

CKD stage subcodes (N18.1–N18.6) are not exposed as matchable variables within the TriNetX propensity interface. Equivalent balance on renal severity was achieved by matching on baseline creatinine, eGFR (MDRD and CKD-EPI 2021), and history of prior AKI, prior metabolic acidosis, and prior hyperkalemia, all direct surrogates of CKD stage. Pre-specified subgroups stratifying by CKD 1–3 vs. CKD 4–6 and a sensitivity analysis excluding dialysis-dependent patients were performed. Patients on chronic dialysis (N18.6 or dialysis procedure codes) were retained in the main analysis and excluded in the “Dialysis Excluded” sensitivity subgroup because an N17 code in a dialysis-dependent patient does not carry the same clinical significance.

### Stage-stratified subgroup analyses and exclusions

Patients carrying only the unspecified CKD code (N18.9) were excluded from all CKD-severity subgroup analyses. This exclusion was pre-specified: because N18.9 does not assign a severity stratum, such patients cannot be allocated to a mild-to-moderate or advanced group without imputation, and their inclusion would contaminate both strata with patients of unknown renal severity. The remaining stage-specified patients were stratified into two mutually exclusive groups mild-to-moderate (N18.1–N18.3) and advanced (N18.4–N18.6) each independently propensity score–matched on the full 61-covariate specification rather than carved out of the already-matched main cohort. Dichotomizing at the stage 3/4 boundary follows the clinical threshold at which eGFR falls below 30 mL/min/1.73 m² and perioperative renal risk rises substantially.

Separately, a pre-specified sensitivity analysis (“Dialysis Excluded”) removed all patients on chronic dialysis and with end-stage renal disease (N18.6 plus dialysis procedure codes), since an N17 code in a dialysis-dependent patient does not carry the same clinical meaning. The AKI association was stronger in this analysis, not weaker (HR 2.146, 95% CI 1.427–3.226; *p* = 0.0002; E-value 3.71, CI 2.21). Patients with ESRD are retained within the advanced stratum, since excluding a stage-specified code would remove the most severe patients from a subgroup defined by severity; the two analyses are complementary rather than overlapping.

Nine pre-specified subgroups were analyzed at the 1-month landmark, each independently matched on all 61 covariates: academic medical centers; age ≥ 65 years; obesity (BMI ≥ 30 kg/m²); CKD stages 1–3; CKD stages 4–6; dialysis excluded; era 2015–2019; era 2020–2026; and hernia type (initial CPT 49650 vs. recurrent CPT 49651). Post-operative eGFR and creatinine were reported for each subgroup.

### Statistical Analysis

Time-to-event outcomes were analyzed by Kaplan–Meier estimation and log-rank testing, with hazard ratios (HRs) and 95% confidence intervals (CIs) from Cox proportional-hazards regression. The proportional-hazards assumption was verified with Schoenfeld residuals; χ² and p-values appear in Table 2. Cox HRs are the primary effect measure in the main text; risk differences, risk ratios, and odds ratios are reserved for the tables. Post-operative laboratory outcomes were compared by independent-sample *t*-test on the most recent value within the follow-up window.

**Table 2:** Clinical Outcomes After Propensity Score Matching

Outcome	Robotic Events/*N*	Lap Events/*N*	Risk Diff	RD 95% CI	HR	HR 95% CI	*p*	Proportionality χ² (*p*)	E-value (CI)
ALL EVENTS (Including patients with outcome prior to time window)									
1- Month Follow up									
MACE	42/1,693	23/1,693	0.011	(0.002, 0.020)	1.843	(1.108, 3.064)	0.017	1.208 (0.272)	3.09 (1.45)
Acute Kidney Injury	84/1,693	49/1,693	0.021	(0.008, 0.034)	1.736	(1.220, 2.469)	0.002	0.061 (0.804)	2.87 (1.74)
Hyperkalemia	32/1,693	30/1,693	0.001	(−0.008, 0.010)	1.072	(0.651, 1.763)	0.787	3.357 (0.067)	1.35 (1)
Metabolic Acidosis	34/1,693	23/1,693	0.006	(−0.002, 0.015)	1.488	(0.877, 2.526)	0.138	1.204 (0.273)	2.34 (1)
Urine Retention	73/1,693	53/1,693	0.012	(−0.001, 0.025)	1.385	(0.972, 1.972)	0.070	0.026 (0.871)	2.12 (1)
6-Month Follow up									
MACE	106/1,659	86/1,659	0.012	(−0.004, 0.028)	1.259	(0.947, 1.673)	0.112	6.292 (0.012)	1.83 (1)
Acute Kidney Injury	182/1,659	136/1,659	0.030	(0.010, 0.049)	1.412	(1.129, 1.766)	0.002	7.730 (0.005)	2.17 (1.51)
Hyperkalemia	77/1,659	96/1,659	−0.014	(−0.030, 0.001)	0.766	(0.569, 1.030)	0.077	4.380 (0.036)	—
Metabolic Acidosis	86/1,659	72/1,659	0.009	(−0.005, 0.023)	1.228	(0.897, 1.682)	0.199	2.562 (0.109)	1.76 (1)
Urine Retention	123/1,659	99/1,659	0.017	(0.001, 0.034)	1.326	(1.014, 1.734)	0.039	1.110 (0.292)	1.98 (1.13)
NEW ONSET ONLY (Excluding patients with outcome prior to time windows									
1- Month Follow up									
Urine Retention ^[1]^	30/1,348	35/1,432	−0.002	(−0.013, 0.009)	0.909	(0.558, 1.480)	0.701	0.063 (0.802)	1.43 (1.00)
6- Month Follow up									
Acute Kidney Injury ^[2]^	54/1,016	37/1,044	0.018	(−0.000, 0.035)	1.515	(0.997, 2.302)	0.052	0.662 (0.416)	2.40 (1.00)
Hyperkalemia ^[3]^	22/1,325	38/1,329	−0.012	(−0.023, −0.001)	0.584	(0.345, 0.987)	0.045	0.313 (0.576)	---
Metabolic Acidosis ^[4]^	43/1,418	34/1,414	0.006	(−0.006, 0.018)	1.274	(0.812, 1.997)	0.291	3.773 (0.052)	1.86 (1.00)
Urine Retention ^[5]^	46/1,329	42/1,415	0.005	(−0.008, 0.018)	1.173	(0.772, 1.782)	0.455	0.367 (0.544)	1.62 (1.00)

Time-to-event clinical outcomes at 1-month and 6-month follow-up after 1:1 propensity score matching, reported under two analytic frames: (i) all-events (including patients with the outcome documented prior to the time window) and (ii) new-onset (excluding patients with any pre-index outcome). Hazard ratios (HR) with 95% confidence intervals (CI) are from Cox proportional-hazards regression; the proportional-hazards assumption was assessed by Schoenfeld residuals (χ²/p reported). E-value and CI E-value are reported for each significant association; the CI E-value applies to the confidence-interval bound nearest the null. A protective effect estimate (HR < 1) yields an undefined E-value and is reported as “—“. Robotic = Cohort 1; Lap = Cohort 2

[1] Urinary retention (new-onset, 1-month): 335 patients in Cohort 1 (robotic) and 251 in Cohort 2 (laparoscopic) were excluded for having an event prior to the time window

[2] Acute kidney injury (new-onset, 6-month): 643 patients in Cohort 1 and 615 in Cohort 2 excluded for prior event

[3] Hyperkalemia (new-onset, 6-month): 334 patients in Cohort 1 and 330 in Cohort 2 excluded for prior event

[4] Metabolic acidosis (new-onset, 6-month): 241 patients in Cohort 1 and 245 in Cohort 2 excluded for prior event

[5] Urinary retention (new-onset, 6-month): 330 patients in Cohort 1 and 244 in Cohort 2 excluded for prior event

Abbreviations: CI, confidence interval; HR, hazard ratio; MACE, major adverse cardiovascular events (composite of acute myocardial infarction [I21], subsequent myocardial infarction [I22], cerebral infarction [I63], or cardiac arrest [I46]); RD, risk difference

E-values were calculated for each significant association from the point HR and the CI bound nearest the null, following VanderWeele and Ding [18]. E-values address unmeasured confounding of an association assumed to be measured without error; they do not speak to differential outcome ascertainment, prevalent-event contamination, or outcome-dependent cohort entry, which are addressed by the new-onset frame, the pre-index CKD timing requirement, and the laboratory-based renal comparison, respectively.

Because TriNetX releases only aggregate summary statistics, individual-level Fine-Gray subdistribution hazard modelling was not feasible; cumulative incidence with death as competing event was approximated from aggregate event and death counts and the Kaplan–Meier survival estimate, following recommended methods for competing-risk analysis in perioperative research [19, 20]. All-cause mortality at 180 days is reported for each arm and the KM–CIF discrepancy is quantified (Fig. 3; Supplementary Figure S1). Given more than 40 comparisons across outcomes, time points, and subgroups, no formal multiplicity correction was applied; secondary and subgroup findings should be considered hypothesis-generating [21, 22]. All tests were two-tailed with *p* < 0.05 considered significant. Analyses used the built-in statistical modules of the TriNetX Analytics Platform.

## Results

### Baseline Characteristics

After 1:1 propensity score matching on 61 pre-specified covariates, the analytical cohorts comprised 1,693 patients per arm at the 1-month landmark and 1,659 per arm at the 6-month landmark (Table 1). The matched cohorts were closely aligned across every major clinical domain. Demographics were comparable between robotic and laparoscopic arms, respectively: mean age 69.9 ± 11.7 vs. 70.4 ± 11.9 years (SMD 0.043) and male sex 89.5% vs. 89.7% (SMD 0.004). Comorbidity burden was equivalent, including essential hypertension (62.7% vs. 61.5%), heart failure (15.7% vs. 15.2%), type 2 diabetes (29.2% vs. 29.2%), dyslipidemia (55.2% vs. 54.6%), and chronic obstructive pulmonary disease (9.6% vs. 9.5%). Cardiovascular history prior acute myocardial infarction (3.4% vs. 3.7%), old myocardial infarction (6.9% vs. 6.5%), and cerebral infarction (2.5% vs. 2.2%) did not differ. CKD-specific matching variables were balanced: prior AKI (10.9% vs. 11.0%), prior metabolic acidosis (3.5% vs. 3.6%), prior hyperkalemia (5.8% vs. 6.4%), baseline creatinine (1.9 ± 4.1 vs. 2.1 ± 6.3 mg/dL), and baseline eGFR MDRD (53.2 ± 21.8 vs. 52.0 ± 22.2 mL/min/1.73 m²; SMD 0.055). Reno-cardiovascular pharmacotherapy including atorvastatin, lisinopril, metoprolol, aspirin, thiazides, and loop diuretics and prior surgical exposures (arterial procedures, CABG, prior hernia repair, colorectal surgery) were similarly distributed, with all SMDs below the 0.10 balance threshold.

A limited number of laboratory summaries showed residual imbalance exceeding the conventional SMD threshold of 0.10 platelet mean volume (0.282), oxygen saturation (0.148), eGFR CKD-EPI 2021 (0.143), and albumin (0.126). These variables were captured in only small sub-populations within the matched cohort, and the reduced denominators rendered perfect covariate matching statistically unachievable within the caliper constraints.

### Clinical Outcomes and Laboratory Results

1-Month Follow-up all-events frame. At 30 days, robotic-assisted repair was associated with a significantly higher incidence of AKI, occurring in 84 of 1,693 patients (5.0%) versus 49 of 1,693 (2.9%) in the laparoscopic arm (risk difference + 2.1%, 95% CI 0.8% to 3.4%; HR 1.736, 95% CI 1.220–2.469; *p* = 0.002; E-value 2.87, CI E-value 1.74). MACE was similarly more frequent with robotic repair (42/1,693 [2.5%] vs. 23/1,693 [1.4%]; HR 1.843, 95% CI 1.108–3.064; *p* = 0.017; E-value 3.09, CI E-value 1.45). Metabolic acidosis (2.0% vs. 1.4%; HR 1.488; *p* = 0.138), urinary retention (4.3% vs. 3.1%; HR 1.385; *p* = 0.070), and hyperkalemia (1.9% vs. 1.8%; HR 1.072; *p* = 0.787) did not differ significantly (Table 2). Post-operative laboratory outcomes at 30 days showed a smaller decline in eGFR MDRD in the robotic arm (Δ − 1.28 vs. − 3.21 mL/min/1.73 m²; t = 1.974, df = 979; *p* = 0.049), whereas eGFR CKD-EPI 2021 (*p* = 0.550) and creatinine (*p* = 0.299) were comparable between arms (Table 3).

**Table 3:** Laboratory Outcomes: Post-operative Values at Follow-up (1-Month & 6-Month)

Lab Marker	Cohort	Baseline Mean ± SD	Post-op Mean ± SD	Mean Difference	t-statistic	df	*p*-value
Main Cohort: 1-Month Follow up							
eGFR MDRD (mL/min/1.73 m²)	Robotic	53.2 ± 21.8	51.925 ± 25.013	−1.275	N\A	N\A	N\A
	Laparoscopic	52.0 ± 22.2	48.788 ± 24.468	−3.212	N\A	N\A	N\A
	Comparison	N\A	N\A	N\A	1.974	979	0.049
eGFR CKD-EPI (mL/min/1.73 m²)	Robotic	55.8 ± 18.4	55.931 ± 18.319	+ 0.131	N\A	N\A	N\A
	Laparoscopic	53.0 ± 20.7	52.742 ± 22.641	−0.258	N\A	N\A	N\A
	Comparison	N\A	N\A	N\A	0.601	64	0.550
Creatinine (mg/dL)	Robotic	1.9 ± 4.1	1.989 ± 2.104	+ 0.089	N\A	N\A	N\A
	Laparoscopic	2.1 ± 6.3	2.130 ± 2.112	+ 0.030	N\A	N\A	N\A
	Comparison	N\A	N\A	N\A	−1.039	983	0.299
Main Cohort: 6 -Month Follow up							
eGFR MDRD (mL/min/1.73 m²)	Robotic	53.2 ± 22.0	53.367 ± 23.026	+ 0.167	N\A	N\A	N\A
	Laparoscopic	52.4 ± 23.2	51.832 ± 24.334	−0.568	N\A	N\A	N\A
	Comparison	N\A	N\A	N\A	1.502	2,146	0.133
eGFR CKD-EPI (mL/min/1.73 m²)	Robotic	57.2 ± 19.2	59.130 ± 20.569	+ 1.930	N\A	N\A	N\A
	Laparoscopic	56.1 ± 20.2	54.071 ± 20.577	−2.029	N\A	N\A	N\A
	Comparison	N\A	N\A	N\A	1.511	153	0.133
Creatinine (mg/dL)	Robotic	1.9 ± 4.1	1.782 ± 1.706	−0.118	N\A	N\A	N\A
	Laparoscopic	1.9 ± 2.0	1.988 ± 2.129	+ 0.088	N\A	N\A	N\A
	Comparison	N\A	N\A	N\A	−2.477	2,155	0.013

Post-operative laboratory outcomes for the main matched cohort at 1-month and 6-month follow-up. Baseline and post-operative values are reported as mean ± SD. Independent-sample t-tests were used for between-arm comparisons of the most recent value within the follow-up window. “Mean Difference” is post-operative minus baseline within each arm. Comparisons across arms are reported with t-statistic, degrees of freedom, and p-value. Abbreviations: CKD-EPI, Chronic Kidney Disease Epidemiology Collaboration; df, degrees of freedom; eGFR, estimated glomerular filtration rate; MDRD, Modification of Diet in Renal Disease; SD, standard deviation

**Fig. 2:**
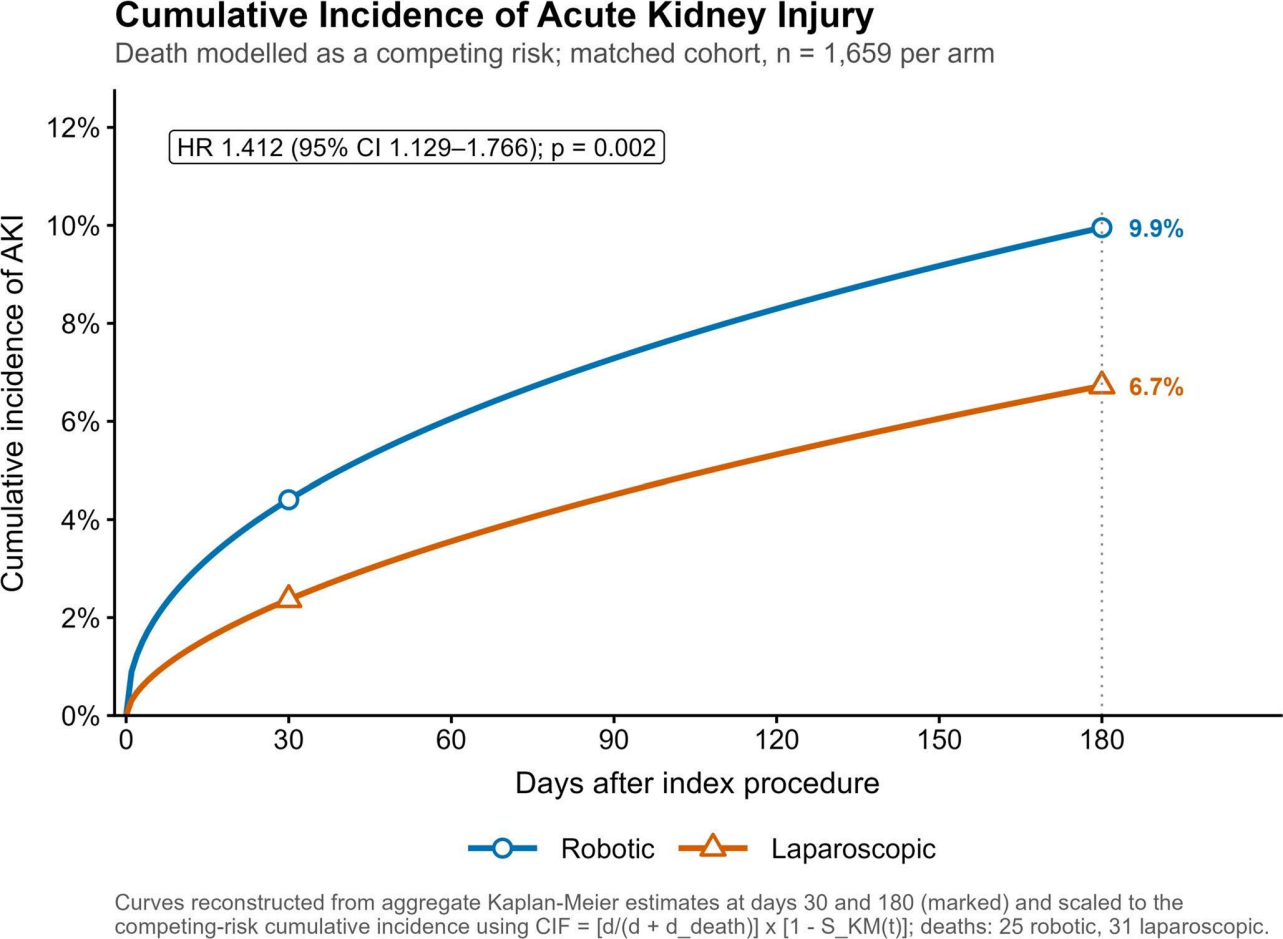
Cumulative Incidence of Acute Kidney Injury (AKI), accounting for death as a competing risk. Cumulative incidence function (CIF) for AKI in robotic-assisted (cyan) versus laparoscopic (red) inguinal hernia repair cohorts, with all-cause death modeled as a competing event. Both arms exhibit a steep early rise during the first 30–60 post-operative days, followed by progressive deceleration after ~ 150 days, with convergence toward a cumulative incidence of ~ 24–25% by the end of observed follow-up. The visual proximity of the two curves confirms that the AKI signal observed in the primary Cox analysis is not an artefact of informative censoring by death

6-Month Follow-up all-events frame. At 180 days, the AKI signal persisted (182/1,659 [11.0%] vs. 136/1,659 [8.2%]; risk difference + 3.0%, 95% CI 1.0% to 4.9%; HR 1.412, 95% CI 1.129–1.766; *p* = 0.002; E-value 2.17, CI E-value 1.51), and urinary retention emerged as a significant late complication (123/1,659 [7.4%] vs. 99/1,659 [6.0%]; HR 1.326, 95% CI 1.014–1.734; *p* = 0.039; E-value 1.98, CI E-value 1.13). The 6-month cumulative-incidence trajectory for AKI, the primary renal endpoint, is depicted in (Fig. 2). Post-operative laboratory outcomes at 6 months showed a lower mean creatinine in the robotic arm (Δ − 0.12 vs. + 0.09 mg/dL; t = − 2.477, df = 2,155; *p* = 0.013), while eGFR MDRD (*p* = 0.133) and eGFR CKD-EPI 2021 (*p* = 0.133) did not differ between arms (Table 3).

The proportional-hazards assumption was met for all 1-month outcomes (all Schoenfeld *p* > 0.05; Table 2). At 6 months, however, the assumption was violated for AKI (χ² = 7.730; *p* = 0.005) and MACE (χ² = 6.292; *p* = 0.012), indicating that the hazard ratio for these outcomes was not constant over the full 180-day window. Visual inspection of the Kaplan-Meier curves for both outcomes showed early separation of the curves during the first 60–90 post-operative days with subsequent convergence, consistent with a time-varying hazard that is highest in the early post-operative period and attenuates thereafter. The reported 6-month HRs of 1.412 (AKI) and 1.259 (MACE) therefore represent time-averaged effects that may understate the early post-operative risk differential and overstate the later differential. The 1-month HRs, which satisfied proportional hazards (AKI: HR 1.736, Schoenfeld *p* = 0.804; MACE: HR 1.843, Schoenfeld *p* = 0.272), more accurately capture the period of maximal risk separation and should be considered the more reliable point estimates. The Kaplan-Meier curves, which make no proportional-hazards assumption, remain valid for visual comparison of cumulative incidence trajectories across the full follow-up period.

6-Month Follow-up new-onset frame. Restricting the analysis to patients without a documented pre-index event isolated two informative signals. Incident AKI trended in the same direction as the overall signal but did not reach conventional significance (HR 1.515, 95% CI 0.997–2.302; *p* = 0.052), indicating that the 6-month AKI excess reflects both new events and post-operative recurrence in patients with a prior AKI history. Conversely, new-onset hyperkalemia was unexpectedly lower in the robotic arm (22/1,325 [1.7%] vs. 38/1,329 [2.9%]; HR 0.584, 95% CI 0.345–0.987; *p* = 0.045); an E-value is not defined for a protective effect, and the finding may reflect differential post-operative fluid and potassium management rather than a biological advantage of robotic surgery.

Across the primary time-to-event endpoints, the 1-month AKI signal was the most robust (CI E-value 1.74): an unmeasured confounder would need to be associated with both surgical approach and AKI by a risk ratio of at least 1.74 beyond the 61 matched covariates to fully explain away the observed effect. The 6-month AKI (CI E-value 1.51) and 1-month MACE (CI E-value 1.45) signals were of comparable moderate robustness, whereas the 6-month urinary-retention signal (CI E-value 1.13) is comparatively more susceptible to residual confounding and should be interpreted with corresponding caution.

### Subgroup Analysis

Subgroup analyses are presented in Supplementary Tables S1–S2 with E-values reported to assess robustness to potential unmeasured confounding. New onset outcomes are not reported due to the scarcity of data available on TriNetx in 1 month follow up duration for all the subgroups.

Age ≥ 65 years. Robotic repair was associated with a significantly higher risk of AKI (HR 1.921, 95% CI 1.235–2.990; *p* = 0.003; E-value 3.25, CI E-value 1.77).

Academic medical centers. The MACE, AKI, and urinary retention signals were amplified in patients treated at academic centers: MACE (HR 3.027, 95% CI 1.530–5.988; *p* = 0.001; E-value 5.50, CI E-value 2.43), AKI (HR 1.647, 95% CI 1.121–2.419; *p* = 0.010; E-value 2.68, CI E-value 1.49), and urinary retention (HR 1.599, 95% CI 1.082–2.364; *p* = 0.018; E-value 2.58, CI E-value 1.38) were all significantly elevated.

Obesity (BMI ≥ 30 kg/m²). AKI was twofold higher in obese patients undergoing robotic repair (HR 1.759, 95% CI 1.041–2.974; *p* = 0.034; E-value 2.91, CI E-value 1.25).

Dialysis-excluded. When patients on chronic dialysis were excluded, effects were most pronounced: MACE (HR 2.397, 95% CI 1.337–4.299; *p* = 0.003; E-value 4.23, CI E-value 2.01), AKI (HR 2.146, 95% CI 1.427–3.226; *p* = 0.0002; E-value 3.71, CI E-value 2.21), and urinary retention (HR 1.968, 95% CI 1.331–2.909; *p* = 0.0006; E-value 3.35, CI E-value 1.99).

Contemporary era (2020–2026). The AKI signal (HR 1.821, 95% CI 1.265–2.622; *p* = 0.0012; E-value 3.04, CI E-value 1.84) and urinary retention signal (HR 1.477, 95% CI 1.027–2.123; *p* = 0.035; E-value 2.32, CI E-value 1.19) persisted in the contemporary era, indicating that the association is not a legacy of early robotic adoption.

Initial (non-recurrent) hernia repair. Robotic repair was associated with significantly higher MACE (HR 2.014, 95% CI 1.177–3.444; *p* = 0.010; E-value 3.44, CI E-value 1.63), AKI (HR 1.764, 95% CI 1.246–2.497; *p* = 0.001; E-value 2.92, CI E-value 1.80), and urinary retention (HR 1.758, 95% CI 1.208–2.557; *p* = 0.003; E-value 2.91, CI E-value 1.71).

Point E-values across subgroups were substantial (2.32–5.50), but several confidence-interval E-values only marginally exceeded 1 most notably for obesity AKI (1.25) and the contemporary-era urinary retention finding (1.19). Because CI E-values quantify the minimum association an unmeasured confounder would need with both exposure and outcome to shift the lower CI bound to the null, values near 1 indicate that even a modest residual confounder could plausibly attenuate these subgroup signals. Accordingly, subgroup findings should be regarded as hypothesis-generating rather than confirmatory and require validation in independent cohorts.

### Competing Risks/All-cause Mortality

**Fig. 3:**
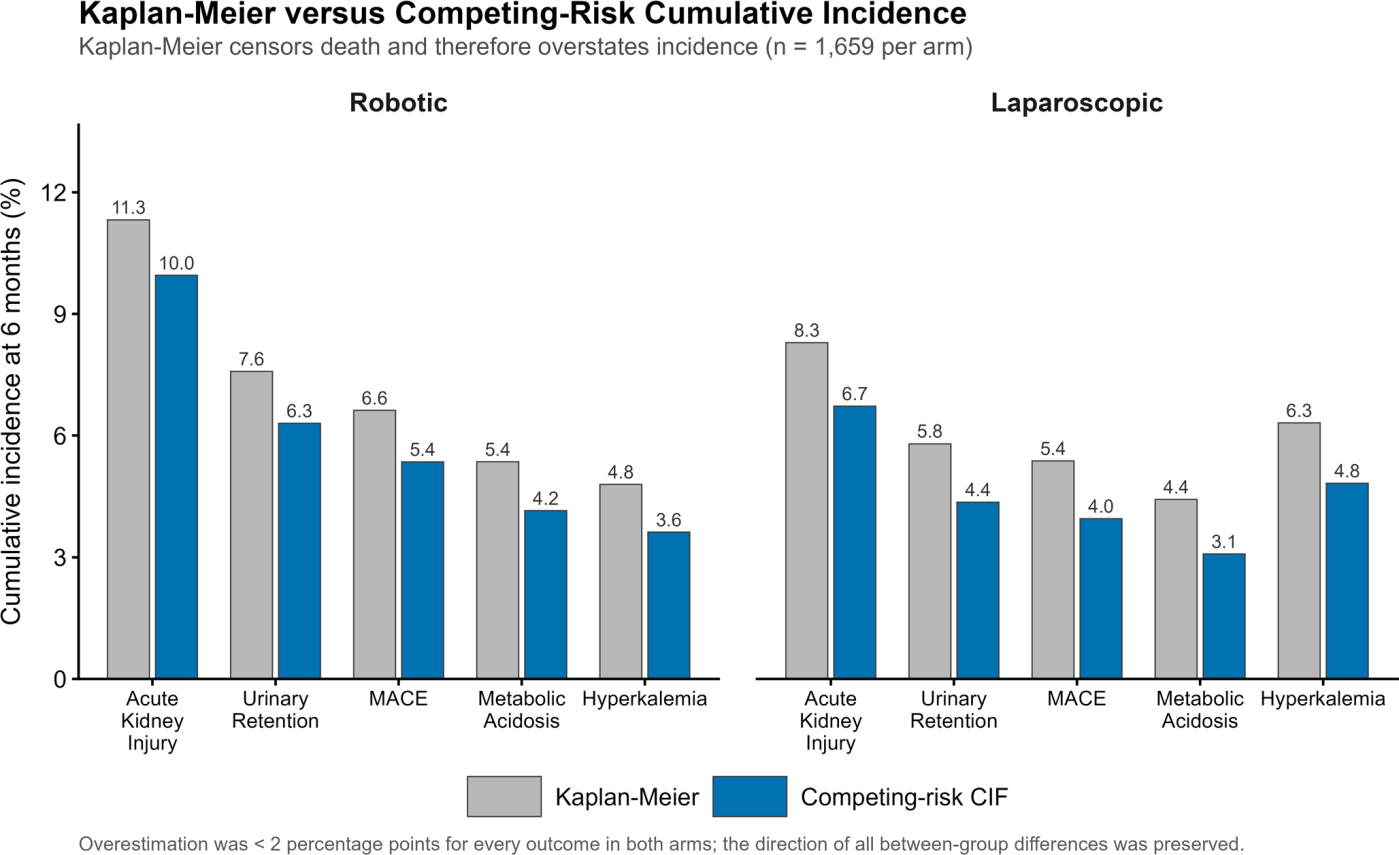
Kaplan-Meier versus Competing-Risk Cumulative Incidence at 6 Months. Head-to-head comparison of Kaplan-Meier (KM) and competing-risk cumulative incidence function (CIF) estimates at 180 days for the five pre-specified outcomes (*N* = 1,659 per arm). Kaplan-Meier estimation systematically overstates absolute incidence relative to CIF because it treats all-cause death (25 [1.5%] robotic; 31 [1.9%] laparoscopic) as non-informative censoring; the direction and rank order of every outcome are preserved under competing-risk correction, confirming that the primary findings are not driven by differential mortality

All-cause mortality within 180 days was low in both arms: 25 of 1,659 patients (1.5%) in the robotic cohort and 31 of 1,659 (1.9%) in the laparoscopic cohort. Because Kaplan-Meier estimation treats death as non-informative censoring, its cumulative-incidence estimates can overstate true risk when a competing mortality risk is present. The KM versus CIF comparison at 6 months (Fig. 3) demonstrated that KM systematically overstated cumulative incidence by approximately 1.1 to 1.9% points across all five outcomes, driven almost entirely by the small competing death rate. After competing-risk adjustment, the AKI CIF curves for the two arms remained separated with the robotic arm carrying the higher cumulative burden throughout follow-up: AKI 10.0% vs. 6.7%, MACE 5.4% vs. 4.0%, urinary retention 6.4% vs. 4.5%, metabolic acidosis 4.2% vs. 3.2%, and hyperkalemia 3.7% (robotic) vs. 4.9% (laparoscopic). Full decomposition of the KM–CIF discrepancy for each outcome is provided in (Supplementary Figure S1). The direction and rank order of every Kaplan-Meier finding were preserved under the competing-risk framework, and the small numerical mortality advantage in the robotic arm did not alter the interpretation, indicating that the primary results are robust to competing-risk adjustment and are not confounded by differential mortality between the two surgical approaches.

## Discussion

In this propensity score matched study of CKD patients across 184 healthcare organizations, to our knowledge, the first CKD-specific comparison of robotic versus laparoscopic inguinal hernia repair, robotic-assisted repair was associated with higher rates of ICD-coded AKI at both 1 month (HR 1.736; *p* = 0.002) and 6 months (HR 1.412; *p* = 0.002) in the all-events analysis, and with higher 1-month MACE (HR 1.843; *p* = 0.017). A modest 6-month excess of urinary retention emerged (HR 1.326; *p* = 0.039), while MACE and metabolic acidosis did not persist as significant at 6 months, hyperkalemia did not differ overall, and new-onset hyperkalemia was unexpectedly lower with robotic repair. However, these ICD-coded findings must be interpreted alongside two countervailing observations: post-operative laboratory eGFR and creatinine trajectories numerically favored the robotic arm, and when the analysis was restricted to incident (new-onset) events, the 6-month AKI signal was directionally consistent but did not reach conventional significance (HR 1.515; *p* = 0.052). Competing-risk cumulative-incidence analysis, which properly accounts for the small mortality differential (1.5% robotic vs. 1.9% laparoscopic), confirmed the direction of the AKI signal while showing that Kaplan-Meier estimation modestly overstated absolute incidence by approximately 1.1–1.9% points.

A central interpretive challenge is the discordance between the ICD-coded AKI signal and the laboratory renal function trajectory. Several non-mutually exclusive explanations merit consideration. First, differential ascertainment is the most parsimonious explanation. Robotic cases were contributed by 49 of 184 queried organizations, whereas laparoscopic cases came from 81, and robotic surgery is concentrated at academic centers with more intensive postoperative laboratory surveillance protocols [23, 24]. More frequent creatinine monitoring increases the probability of detecting transient elevations that trigger N17 coding, even when the overall renal trajectory is favorable. This interpretation is supported by the higher proportion of robotic patients with documented eGFR at 1 month and is consistent with evidence that ICD-10 AKI coding has high positive predictive value (91%) but very low sensitivity (7.5%), with sensitivity varying substantially by institution, AKI severity, and clinical context [25, 26]. In a Danish validation study of nearly 1 million laboratory-identified AKI episodes, only 4% of stage 1 AKI episodes were captured by ICD-10 codes, compared with 22% of stage 3 episodes, indicating that coding is heavily influenced by clinical recognition and documentation practices rather than by a uniform biochemical threshold [25]. If robotic centers code mild, self-limited creatinine elevations more frequently than community laparoscopic sites, the ICD-coded AKI difference could be an artefact of differential surveillance rather than a true difference in renal injury.

Moreover, the ICD-10 N17 code captures a heterogeneous spectrum from transient, clinically insignificant creatinine rises to severe AKI requiring renal replacement therapy [27, 28]. The robotic arm’s excess coded events may reflect milder, self-limited episodes consistent with the pneumoperitoneum-induced transient oliguria and creatinine elevation that typically resolves within 24 h of desufflation that do not alter the aggregate eGFR trajectory [29, 30]. The severity, duration, and reversibility of coded AKI episodes cannot be determined from administrative data, and non-verified ICD-10 complication codes have been shown to significantly overestimate in-hospital surgical complication rates [31, 32]. 

If the association between robotic repair and AKI reflects a true causal effect, it is biologically plausible. Pneumoperitoneum at intra-abdominal pressures above 10 mmHg reduces renal blood flow through direct compression, carbon dioxide absorption, neuroendocrine activation, and oxidative stress, with pre-existing kidney disease amplifying these effects [30]. A systematic review confirmed reduced renal perfusion and function during pneumoperitoneum, scaling with pressure, duration, and preoperative renal status [33]. Robotic repair entails consistently longer operative times, approximately 14 min longer in one meta-analysis and 35 min longer in the RIVAL trial, raising the pneumoperitoneum “exposure index” (pressure × time), itself an independent predictor of post-laparoscopic AKI (OR 1.47, 95% CI 1.05–2.04) [34–36]. In diminished renal reserve, this prolonged insult may cross a threshold for injury, as shown experimentally in rats with chronic kidney injury, while the Trendelenburg position used in robotic surgery may further compromise renal hemodynamics [37, 38]. However, these mechanisms remain speculative in the present context, as operative time, pneumoperitoneum pressure, and intraoperative hemodynamics were not available in the TriNetX database.

Subgroup analyses should be regarded as exploratory and hypothesis-generating, as no formal multiplicity correction was applied across the > 40 comparisons. The AKI signal was directionally consistent across most subgroups, with the strongest effects observed when patients on chronic dialysis were excluded (HR 2.146; *p* = 0.0002; E-value 3.71, CI E-value 2.21) and in the contemporary era 2020–2026 (HR 1.821; *p* = 0.0012), suggesting the association is not a legacy of early robotic adoption. The elderly subgroup (≥ 65 years) showed a higher AKI risk (HR 1.921; *p* = 0.003), consistent with the known increase in perioperative cardiac and renal risk across CKD stages [27]. 

### Comparison with the Broader Literature

Prior comparisons of robotic versus laparoscopic inguinal hernia repair examined general populations and found comparable safety. The RIVAL randomized trial the first level-I trial showed no difference in wound events, readmissions, pain, or quality of life through 2 years but did not enroll CKD patients or assess renal outcomes [35, 39]. The ROGER randomized trial (*N* = 182) similarly found no difference in postoperative pain or complication rates between robotic TAPP and laparoscopic TEP, while confirming longer operative times with robotics (+ 16 min for unilateral repairs); renal outcomes were not assessed [40]. The ROLAIS trial reported a lower inflammatory response and fewer complications with robotic TAPP (23.0% vs. 41.5%; *p* = 0.029) but did not stratify by renal function or report renal endpoints [41]. Meta-analyses by Li et al. (OR 1.22, 95% CI 0.68–2.18) and de’Angelis et al. found comparable complication profiles without renal-specific endpoints, and a 153,727-patient New York study found no perioperative differences after matching, again without CKD stratification [34, 42, 43]. With 56% of major cardiovascular trials excluding renal patients, the present study addresses a systematic evidence gap [44]. However, the discordance between ICD-coded and laboratory-based renal outcomes in the present analysis means that the question of whether safety equivalence in general populations extends to CKD remains open rather than answered.

### Strengths and Limitations

Strengths include the first renal and cardiovascular comparison specific to CKD, a large multi-institutional matched cohort (3,386 patients at 1 month), matching on 61 covariates including prior AKI, prior metabolic acidosis, prior hyperkalemia, and baseline eGFR, dual analytic frames, competing-risk analysis with all-cause mortality, E-value sensitivity analysis, nine independently matched subgroups, and post-operative laboratory renal function as a coding-independent comparator.

Several limitations must be acknowledged. First, the retrospective design cannot exclude residual confounding from unmeasured factors including operative time, pneumoperitoneum pressure, intra-operative hemodynamics, surgeon experience, institutional robotic case volume, technique (TAPP vs. TEP), anesthetic management, bilateral vs. unilateral repair, hernia complexity, prior abdominal surgery, and frailty [28, 36]. Second, the primary renal outcome relies on ICD-10 N17 rather than KDIGO-defined AKI; ICD-10 AKI codes have high positive predictive value but very low sensitivity, and coding practices may differ systematically across the 184 participating HCOs. The discordance between the coded signal and the favorable laboratory trajectory in the robotic arm is the most important interpretive limitation. Third, calendar year of surgery and site-level characteristics are not available as matchable covariates in TriNetX; era-stratified and academic-center subgroup analyses partially but do not fully resolve temporal and institutional confounding, including the secular rise in AKI coding intensity with wider KDIGO adoption. Fourth, more than 40 comparisons were performed without multiplicity correction; the primary all-events AKI finding would survive Bonferroni correction, but the new-onset AKI (*p* = 0.052), 1-month MACE (*p* = 0.017), and subgroup findings should be considered hypothesis-generating. Fifth, four laboratory covariates showed residual post-matching SMD > 0.10 (platelet mean volume 0.282; oxygen saturation 0.148; eGFR CKD-EPI 2021 0.143; albumin 0.126), reflecting small denominators; five additional specialized covariates (eGFR CKD-EPI 2021, urine albumin/creatinine, troponin I, BNP, NT-proBNP) had baseline availability below 10% and cannot be imputed because TriNetX releases only aggregate data. Balance on the widely available covariates most relevant to CKD physiology provides the substantive basis for exchangeability. Although the distribution of individual CKD stages was well balanced both before and after matching (Table 1; maximum stage-specific SMD 0.074 before and 0.012 after), residual confounding by CKD stage cannot be entirely excluded, since 36% of patients in each arm carried only an unspecified CKD code and could not be assigned to a severity stratum. Sixth, TriNetX aggregate-only output precluded individual-level Fine-Gray modelling; CIF was approximated from aggregate event and death counts. The platform cannot ascertain cause-specific mortality, and the predominantly US network may limit generalizability. Recent meta-research has questioned methodological rigor in some TriNetX studies, though transparent index-event definitions and the dual-frame analysis mitigate several identified concerns [45]. Seventh, the proportional-hazards assumption was violated for 6-month AKI and MACE; the 6-month HRs represent time-averaged effects, and the 1-month estimates more reliably capture the period of maximal risk separation. Piecewise or time-varying Cox models were not feasible within the built-in TriNetX analytic modules.

### Clinical Implications and Future Directions

The results do not argue against robotic surgery broadly but reinforce the importance of population-specific risk assessment in vulnerable groups. When robotic repair is chosen in CKD, renal-protective measures are advisable minimizing pneumoperitoneum pressure and duration, optimizing hemodynamics and hydration, avoiding nephrotoxins, and maintaining systolic blood pressure near the patient’s usual level [28, 46]. Prospective validation is required before these findings should influence approach selection.

Future work should prioritize prospective or randomized comparisons in CKD with granular intra-operative data (operative time, pneumoperitoneum parameters, hemodynamics, fluid balance); KDIGO-defined AKI using paired pre- and post-operative creatinine, which would resolve the coding-versus-laboratory discordance observed here; biomarkers of subclinical AKI such as NGAL and KIM-1; preoperative renal functional-reserve assessment for risk stratification; renoprotective low-pressure pneumoperitoneum protocols; and independent, multiplicity-adjusted validation of the secondary findings [36]. 

## Conclusion

In this propensity score–matched cohort of 3,386 CKD patients undergoing minimally invasive inguinal hernia repair, robotic-assisted repair was associated with higher rates of ICD-coded AKI and early MACE in the all-events analysis, but the incident AKI signal was attenuated when restricted to new-onset events (HR 1.515; *p* = 0.052), and post-operative eGFR and creatinine trajectories numerically favored the robotic arm. This discordance between administrative coding and laboratory-based renal function represents the central interpretive limitation of this study and may reflect differential surveillance and coding practices at robotic-predominant academic centers. Competing-risk analysis accounting for the small mortality signal confirmed the direction of the coded AKI excess without evidence of differential mortality. Whether conventional laparoscopy confers a true renal safety advantage over robotic repair in CKD therefore remains unresolved: the present data are consistent both with a genuine biological effect of prolonged pneumoperitoneum and with a surveillance artefact arising from more intensive laboratory monitoring in the robotic arm. Until prospective data with KDIGO-defined endpoints are available, the choice of minimally invasive approach in CKD should be individualized rather than dictated by these findings. These findings are hypothesis-generating; prospective validation using KDIGO-defined AKI with paired pre- and post-operative creatinine values is needed before they should influence surgical approach selection.

## Supplementary Information

Below is the link to the electronic supplementary material.

Supplementary Material 1

Supplementary Material 2

Supplementary Material 3

Supplementary Material 4

## Data Availability

Data were obtained under license from the TriNetX research network and are not publicly available, but are available from the authors upon reasonable request and with permission of TriNetX.

## Abbreviations

AKI: acute kidney injury
CIF: cumulative incidence function
CI: confidence interval
CKD: chronic kidney disease
COPD: chronic obstructive pulmonary disease
CPT: Current Procedural Terminology
eGFR: estimated glomerular filtration rate
EHR: electronic health record
HCO: healthcare organization
HIPAA: Health Insurance Portability and Accountability Act
HR: hazard ratio
ICD-10-CM: International Classification of Diseases, Tenth Revision, Clinical Modification
ICD-10-PCS: ICD-10 Procedure Coding System
IRB: institutional review board
KDIGO: Kidney Disease: Improving Global Outcomes
KM: Kaplan-Meier
LOINC: Logical Observation Identifiers Names and Codes
MACE: major adverse cardiovascular events
MDRD: Modification of Diet in Renal Disease
NKF-ASN: National Kidney Foundation–American Society of Nephrology
OR: odds ratio
PSM: propensity score matching
SMD: standardized mean difference
SNOMED: Systematized Nomenclature of Medicine Clinical Terms
STROBE: Strengthening the Reporting of Observational Studies in Epidemiology
TAPP: transabdominal preperitoneal
TEP: totally extraperitoneal

## Declarations

### Declaration of AI Use

In accordance with the journal’s AIGC policy and the COPE position statement, the authors declare the use of Gemini to assist in producing the graphical abstract. The tool was not used as an author and had no role in study design or data interpretation; the authors take full responsibility for the figure’s accuracy and final content.
