# Supplementary material for: Robotic vs. laparoscopic inguinal hernia repair in patients with chronic kidney disease: a propensity score-matched analysis
Source: J Robot Surg. 2026 Aug 18;20(1):850. doi: 10.1007/s11701-026-03782-y (PMC13486028; doi:10.1007/s11701-026-03782-y)
Supplement: Supplementary file 1 [file 11701_2026_3782_MOESM1_ESM.docx]

**STROBE Statement — Checklist of Items for Reports of Cohort Studies**

*Robotic vs. Laparoscopic Inguinal Hernia Repair in Patients with Chronic Kidney Disease: A Propensity Score–Matched Analysis of the TriNetX Global Database*

| **Section** | **No.** | **STROBE Recommendation** | **Reported on page / section** |
| --- | --- | --- | --- |
| Title and abstract | **1(a)** | Indicate the study's design with a commonly used term in the title or the abstract. | Title (page 1) 'A Propensity Score–Matched Analysis'; Abstract Methods paragraph. |
| Title and abstract | **1(b)** | Provide in the abstract an informative and balanced summary of what was done and what was found. | Abstract: Background / Methods / Results / Conclusions paragraphs (page 1). |
| **Introduction** |  |  |  |
| Background / rationale | **2** | Explain the scientific background and rationale for the investigation being reported. | Introduction, paragraphs 1–2. |
| Objectives | **3** | State specific objectives, including any prespecified hypotheses. | Introduction, final paragraph (study objective statement). |
| **Methods** |  |  |  |
| Study design | **4** | Present key elements of study design early in the paper. | Methods 'Study Design' subsection. |
| Setting | **5** | Describe the setting, locations, and relevant dates, including periods of recruitment, exposure, follow-up, and data collection. | Methods 'Data Source' and 'Study Population and Cohort Definitions' subsections; date window 1 May 2015 – 1 May 2026; analysis date July 2026. |
| Participants | **6(a)** | Give the eligibility criteria, and the sources and methods of selection of participants. Describe methods of follow-up. | Methods 'Study Population and Cohort Definitions'; Figure 1 (cohort flow diagram). |
| Participants | **6(b)** | For matched studies, give matching criteria and number of exposed and unexposed. | Methods 'Propensity Score Matching' subsection; Table 1 (61 matched covariates); N = 1,693 per arm at 1 month; N = 1,659 per arm at 6 months. |
| Variables | **7** | Clearly define all outcomes, exposures, predictors, potential confounders, and effect modifiers. Give diagnostic criteria if applicable. | Methods 'Outcomes' subsection (ICD-10, LOINC, CPT/SNOMED codes) and 'Propensity Score Matching' (covariates). |
| Data sources / measurement | **8*** | For each variable of interest, give sources of data and details of methods of assessment. Describe comparability of assessment methods if more than one group. | Methods 'Data Source' and 'Outcomes' subsections; codes and LOINC/TNX identifiers specified. |
| Bias | **9** | Describe any efforts to address potential sources of bias. | Methods dual analytic frame (all-events vs. new-onset); pre-index CKD timing requirement; era-stratified and academic-center subgroups; competing-risk CIF; E-values. Limitations differential ascertainment, coding variability. |
| Study size | **10** | Explain how the study size was arrived at. | Determined by all TriNetX patients meeting inclusion criteria within the pre-specified index window; no a priori sample-size calculation (Methods — 'Study Population'). |
| Quantitative variables | **11** | Explain how quantitative variables were handled in the analyses. If applicable, describe which groupings were chosen and why. | Methods 'Statistical Analysis'; baseline laboratory variables retained continuous for matching; CKD-stage subgroups pre-specified as 1–3 vs. 4–6; age subgroup ≥65 years pre-specified. |
| Statistical methods | **12(a)** | Describe all statistical methods, including those used to control for confounding. | Methods Statistical Analysis' and 'Propensity Score Matching' subsections. |
| Statistical methods | **12(b)** | Describe any methods used to examine subgroups and interactions. | Methods 'Subgroup Analyses' subsection; each subgroup independently matched on the full 61-covariate specification. |
| Statistical methods | **12(c)** | Explain how missing data were addressed. | Methods 'Handling of missing laboratory data' subsection; complete-case within-covariate approach; Supplementary Table S-Missing reports availability of every baseline laboratory covariate; no imputation performed. |
| Statistical methods | **12(d)** | If applicable, explain how loss to follow-up was addressed. | Methods 'Statistical Analysis'; censoring at last recorded clinical fact for KM/Cox; CIF treats all-cause death as competing event. |
| Statistical methods | **12(e)** | Describe any sensitivity analyses. | Methods E-value analysis, new-onset analytic frame, dialysis-excluded subgroup, era-stratified subgroups, academic-center subgroup, initial vs. recurrent hernia subgroup. |
| **Results** |  |  |  |
| Participants | **13(a)** | Report numbers of individuals at each stage of study. | Figure 1 (cohort flow diagram) with counts at every step; Results 'Baseline Characteristics' paragraph. |
| Participants | **13(b)** | Give reasons for non-participation at each stage. | Figure 1 CKD required ≥1 month before index (n = 10,068 excluded); unmatched patients displayed (15 robotic in 1-month cohort; 4 robotic in 6-month cohort). |
| Participants | **13(c)** | Consider use of a flow diagram. | Figure 1 (Cohort Flow Diagram). |
| Descriptive data | **14(a)** | Give characteristics of study participants and information on exposures and potential confounders. | Table 1: 61-covariate baseline characteristics before and after matching. |
| Descriptive data | **14(b)** | Indicate number of participants with missing data for each variable of interest. | Supplementary Table S-Missing availability of every baseline laboratory covariate before and after matching. |
| Descriptive data | **14(c)** | Summarize follow-up time. | Results mean follow-up 29.4 days (1-month cohort) and 179 days (6-month cohort); reported in Methods 'Study Population and Cohort Definitions'. |
| Outcome data | **15*** | Report numbers of outcome events or summary measures over time. | Table 2 (event counts and rates for all clinical outcomes at 1 and 6 months); Table 3 (post-operative laboratory outcomes); Figure 2 (AKI CIF); Figure 3 (KM vs. CIF). |
| Main results | **16(a)** | Give unadjusted estimates and, if applicable, confounder-adjusted estimates and their precision. | Table 2 (risk differences, risk ratios, odds ratios, hazard ratios with 95% CIs); Results 'Clinical Outcomes and Laboratory Results' subsection. |
| Main results | **16(b)** | Report category boundaries when continuous variables were categorized. | Methods 'Subgroup Analyses' (age ≥65; BMI ≥30 kg/m²; CKD 1–3 vs. 4–6; era 2015–2019 vs. 2020–2026). |
| Main results | **16(c)** | If relevant, consider translating estimates of relative risk into absolute risk for a meaningful time period. | Table 2: risk differences reported alongside HRs; Results give absolute event proportions at 30 and 180 days for every outcome. |
| Other analyses | **17** | Report other analyses done subgroups and interactions, and sensitivity analyses. | Supplementary Tables S1–S2 (nine independently matched subgroups); Results 'Subgroup Analyses' subsection; E-values reported for every significant association. |
| **Discussion** |  |  |  |
| Key results | **18** | Summarize key results with reference to study objectives. | Discussion, opening paragraph. |
| Limitations | **19** | Discuss limitations of the study, taking into account sources of potential bias or imprecision. | Discussion 'Strengths and Limitations' subsection (seven numbered limitations including differential ascertainment, ICD-10 coding limitations, unmatched calendar year, multiplicity, residual SMD imbalance, aggregate-only TriNetX data, and violated proportional-hazards for 6-month outcomes). |
| Interpretation | **20** | Give a cautious overall interpretation considering objectives, limitations, multiplicity, results from similar studies, and other relevant evidence. | Discussion 'The central discordance,' 'Biological plausibility,' 'Comparison with the broader literature'; Conclusion 'hypothesis-generating' framing. |
| Generalisability | **21** | Discuss the generalizability (external validity) of the study results. | Discussion 'Strengths and Limitations' (predominantly US network; academic-center concentration of robotic cases; unmeasured procedural factors). |
| **Other information** |  |  |  |
| Funding | **22** | Give the source of funding and the role of the funders. | Declarations 'Funding: No funding was received for this study.' |

**Give information separately for exposed and unexposed groups. An Explanation & Elaboration article discusses each checklist item and gives methodological background and published examples of transparent reporting: PLoS Medicine at http://www.plosmedicine.org/ and Annals of Internal Medicine at http://www.annals.org/. Information on the STROBE Initiative is available at www.strobe-statement.org.*
