# Supplementary material for: Robotic vs. laparoscopic inguinal hernia repair in patients with chronic kidney disease: a propensity score-matched analysis
Source: J Robot Surg. 2026 Aug 18;20(1):850. doi: 10.1007/s11701-026-03782-y (PMC13486028; doi:10.1007/s11701-026-03782-y)
Supplement: Supplementary file 2 [file 11701_2026_3782_MOESM2_ESM.docx]

**Supplementary File 1**

*Query Specifications, Index Event Definitions, and Outcome Ascertainment (TriNetX Global Collaborative Network)*

### Data Source and Extraction Dates

All analyses were performed on the TriNetX Global Collaborative Network. The cohort query was executed on May 29, 2026, at which point 184 healthcare organization(s) (HCOs) were queried and 184 HCO(s) responded. All comparative analyses reported in this manuscript were executed between July 20 and July 21, 2026. Robotic inguinal hernia repair cases were contributed by 49 responding HCOs, whereas conventional laparoscopic cases were contributed by 81 HCOs.

### Query Criteria for Cohort 1 (query name: Robotic Final )

This query was run on the network Global Collaborative Network with 184 HCO(s) queried and 184 HCO(s) responded. A total of 49 provider(s) responded with patients. The final cohort included 1,708 patients who matched the query criteria listed in the table below. Chronic kidney disease (ICD-10-CM N18) was required to be documented at least 1 month before the index procedure. For the text representation of the query criteria please see Appendix A.

| Group 1 or Group 2 must be present | | | | | |
| --- | --- | --- | --- | --- | --- |
| Group 1 | | | | | |
|  | **Group 1A Robotic** | | | | |
|  | must have | any of | procedure | UMLS:CPT:49650 | Laparoscopy, surgical; repair initial inguinal hernia |
|  |  |  | procedure | UMLS:CPT:49651 | Laparoscopy, surgical; repair recurrent inguinal hernia |
|  |  |  | procedure | UMLS:SNOMED:15018006 | Laparoscopic repair of inguinal hernia |
|  | date constraint | | The terms in this group occurred between May 1, 2015 and May 1, 2026 | | |
|  | event relationship | | Any instance of Group 1B occurred within 1 day on or after any instance of Robotic | | |
|  | **Group 1B** | | | | |
|  | must have | any of | procedure | UMLS:HCPCS:S2900 | Surgical techniques requiring use of robotic surgical system (list separately in addition to code for primary procedure) |
|  |  |  | procedure | UMLS:ICD10PCS:8E0W4CZ | Robotic Assisted Procedure of Trunk Region, Percutaneous Endoscopic Approach |
| Group 2 | | | | | |
|  | **Group 2A** | | | | |
|  | must have | any of | procedure | UMLS:SNOMED:1156323002 | Repair of right inguinal hernia using robotic assistance |
|  |  |  | procedure | UMLS:SNOMED:1156322007 | Repair of inguinal hernia using robotic assistance |
|  |  |  | procedure | UMLS:SNOMED:1156324008 | Repair of left inguinal hernia using robotic assistance |
|  | date constraint | | The terms in this group occurred between May 1, 2015 and May 1, 2026 | | |
| Group 3 | | | | | |
|  | **Group 3A Robotic** | | | | |
|  | must have | any of | procedure | UMLS:CPT:49650 | Laparoscopy, surgical; repair initial inguinal hernia |
|  |  |  | procedure | UMLS:CPT:49651 | Laparoscopy, surgical; repair recurrent inguinal hernia |
|  |  |  | procedure | UMLS:SNOMED:15018006 | Laparoscopic repair of inguinal hernia |
|  |  |  | procedure | UMLS:ICD10PCS:8E0W4CZ | Robotic Assisted Procedure of Trunk Region, Percutaneous Endoscopic Approach |
|  |  |  | procedure | UMLS:HCPCS:S2900 | Surgical techniques requiring use of robotic surgical system (list separately in addition to code for primary procedure) |
|  |  |  | procedure | UMLS:SNOMED:1156323002 | Repair of right inguinal hernia using robotic assistance |
|  |  |  | procedure | UMLS:SNOMED:1156322007 | Repair of inguinal hernia using robotic assistance |
|  |  |  | procedure | UMLS:SNOMED:1156324008 | Repair of left inguinal hernia using robotic assistance |
|  | date constraint | | The terms in this group occurred at any time | | |
|  | event relationship | | Any instance of CKD occurred at least 1 month before any instance of Robotic | | |
|  | **Group 3B CKD** | | | | |
|  | must have |  | diagnosis | UMLS:ICD10CM:N18 | Chronic kidney disease (CKD) |

### Query Criteria for Cohort 2 (query name: Laparoscopy Final)

This query was run on the network Global Collaborative Network with 184 HCO(s) queried and 184 HCO(s) responded. A total of 81 provider(s) responded with patients. The final cohort included 6,853 patients who matched the query criteria listed in the table below. Chronic kidney disease (ICD-10-CM N18) was required to be documented at least 1 month before the index procedure.

| Group 1 | | | | | |
| --- | --- | --- | --- | --- | --- |
|  | **Group 1A Robotic** | | | | |
|  | must have | any of | procedure | UMLS:CPT:49650 | Laparoscopy, surgical; repair initial inguinal hernia |
|  |  |  | procedure | UMLS:CPT:49651 | Laparoscopy, surgical; repair recurrent inguinal hernia |
|  |  |  | procedure | UMLS:SNOMED:15018006 | Laparoscopic repair of inguinal hernia |
|  | date constraint | | The terms in this group occurred between May 1, 2015 and May 1, 2026 | | |
|  | event relationship | | Any instance of Group 1B occurred within 1 day on or after any instance of Robotic | | |
|  | **Group 1B** | | | | |
|  | cannot have |  | procedure | UMLS:ICD10PCS:8E0W4CZ | Robotic Assisted Procedure of Trunk Region, Percutaneous Endoscopic Approach |
|  |  | or | procedure | UMLS:HCPCS:S2900 | Surgical techniques requiring use of robotic surgical system (list separately in addition to code for primary procedure) |
| Group 2 | | | | | |
|  | **Group 2A Robotic** | | | | |
|  | must have | any of | procedure | UMLS:CPT:49650 | Laparoscopy, surgical; repair initial inguinal hernia |
|  |  |  | procedure | UMLS:CPT:49651 | Laparoscopy, surgical; repair recurrent inguinal hernia |
|  |  |  | procedure | UMLS:SNOMED:15018006 | Laparoscopic repair of inguinal hernia |
|  | cannot have |  | procedure | UMLS:SNOMED:1156324008 | Repair of left inguinal hernia using robotic assistance |
|  |  | or | procedure | UMLS:HCPCS:S2900 | Surgical techniques requiring use of robotic surgical system (list separately in addition to code for primary procedure) |
|  |  | or | procedure | UMLS:SNOMED:1156322007 | Repair of inguinal hernia using robotic assistance |
|  |  | or | procedure | UMLS:SNOMED:1156323002 | Repair of right inguinal hernia using robotic assistance |
|  |  | or | procedure | UMLS:SNOMED:1156322007 | Repair of inguinal hernia using robotic assistance |
|  |  | or | procedure | UMLS:SNOMED:1156324008 | Repair of left inguinal hernia using robotic assistance |
|  |  | or | procedure | UMLS:SNOMED:1156323002 | Repair of right inguinal hernia using robotic assistance |
|  |  | or | procedure | UMLS:ICD10PCS:8E0W4CZ | Robotic Assisted Procedure of Trunk Region, Percutaneous Endoscopic Approach |
|  | date constraint | | The terms in this group occurred at any time | | |
|  | event relationship | | Any instance of CKD occurred at least 1 month before any instance of Robotic | | |
|  | **Group 2B CKD** | | | | |
|  | must have |  | diagnosis | UMLS:ICD10CM:N18 | Chronic kidney disease (CKD) |

### Index Events Used in this Analysis

Index events for the Compare Outcomes analysis were derived from the cohort definitions. Index events were defined separately for each cohort and were based on the criteria used in the original cohort definition. The index event was the first qualifying inguinal hernia repair meeting the respective cohort definition. Please see Appendix B for the text representation of the index event definitions.

The index event for Cohort 1 (query name: Robotic Final ) was defined as the following:

| Group 1 or Group 2 must be present | | | | | |
| --- | --- | --- | --- | --- | --- |
| Group 1 | | | | | |
|  | **Group 1A Robotic** | | | | |
|  | must have | any of | procedure | UMLS:CPT:49650 | Laparoscopy, surgical; repair initial inguinal hernia |
|  |  |  | procedure | UMLS:CPT:49651 | Laparoscopy, surgical; repair recurrent inguinal hernia |
|  |  |  | procedure | UMLS:SNOMED:15018006 | Laparoscopic repair of inguinal hernia |
|  | date constraint | | The terms in this group occurred between May 1, 2015 and May 1, 2026 | | |
|  | event relationship | | Any instance of Group 1B occurred within 1 day on or after any instance of Robotic | | |
|  | **Group 1B** | | | | |
|  | must have | any of | procedure | UMLS:HCPCS:S2900 | Surgical techniques requiring use of robotic surgical system (list separately in addition to code for primary procedure) |
|  |  |  | procedure | UMLS:ICD10PCS:8E0W4CZ | Robotic Assisted Procedure of Trunk Region, Percutaneous Endoscopic Approach |
| Group 2 | | | | | |
|  | **Group 2A** | | | | |
|  | must have | any of | procedure | UMLS:SNOMED:1156323002 | Repair of right inguinal hernia using robotic assistance |
|  |  |  | procedure | UMLS:SNOMED:1156322007 | Repair of inguinal hernia using robotic assistance |
|  |  |  | procedure | UMLS:SNOMED:1156324008 | Repair of left inguinal hernia using robotic assistance |
|  | date constraint | | The terms in this group occurred between May 1, 2015 and May 1, 2026 | | |

The index event for Cohort 2 (query name: Laparoscopy Final) was defined as the following:

| Group 1 | | | | | |
| --- | --- | --- | --- | --- | --- |
|  | **Group 1A Robotic** | | | | |
|  | must have | any of | procedure | UMLS:CPT:49650 | Laparoscopy, surgical; repair initial inguinal hernia |
|  |  |  | procedure | UMLS:CPT:49651 | Laparoscopy, surgical; repair recurrent inguinal hernia |
|  |  |  | procedure | UMLS:SNOMED:15018006 | Laparoscopic repair of inguinal hernia |
|  | date constraint | | The terms in this group occurred between May 1, 2015 and May 1, 2026 | | |
|  | event relationship | | Any instance of Group 1B occurred within 1 day on or after any instance of Robotic | | |
|  | **Group 1B** | | | | |
|  | cannot have |  | procedure | UMLS:ICD10PCS:8E0W4CZ | Robotic Assisted Procedure of Trunk Region, Percutaneous Endoscopic Approach |
|  |  | or | procedure | UMLS:HCPCS:S2900 | Surgical techniques requiring use of robotic surgical system (list separately in addition to code for primary procedure) |

### Time Windows Used in this Analysis

Two pre-specified landmarks were analyzed. The primary 1-month analysis included outcomes that occurred in the time window that started 1 day after the first occurrence of the index event and ended 30 days after the first occurrence of the index event, with an index window of May 1, 2015 to May 1, 2026. The secondary 6-month analysis included outcomes that occurred in the time window that started 1 day after and ended 180 days after the index event; its index window was restricted to May 1, 2015 to January 1, 2026 so that every included patient had at least 180 days of potential follow-up before the extraction date. Outcome ascertainment began at day 1 rather than day 0 because TriNetX assigns procedure and diagnosis codes to the same calendar date, and including day 0 would risk capturing diagnoses documented during the index encounter itself rather than true postoperative events.

### Outcome Definitions

The table below outlines the definitions for each outcome and the analysis specifications. For outcome definitions consisting of more than one term, at least one term must match. Please see Appendix C for the text representation of the outcome definitions.

| MACE | | | | |
| --- | --- | --- | --- | --- |
|  | **Outcome definition** | | | |
|  | | Diagnosis | UMLS:ICD10CM:I21 | Acute myocardial infarction |
|  | | Diagnosis | UMLS:ICD10CM:I22 | Subsequent ST elevation (STEMI) and non-ST elevation (NSTEMI) myocardial infarction |
|  | | Diagnosis | UMLS:ICD10CM:I63 | Cerebral infarction |
|  | | Diagnosis | UMLS:ICD10CM:I46 | Cardiac arrest |
|  | **Settings for the performed analyses** | | | |
|  | | Risk analysis | | including patients with outcome prior to the time window |
|  | | Kaplan - Meier survival analysis | | including patients with outcome prior to the time window |
| Acute Kidney Injury | | | | |
|  | **Outcome definition** | | | |
|  | | Diagnosis | UMLS:ICD10CM:N17 | Acute kidney failure |
|  | **Settings for the performed analyses** | | | |
|  | | Risk analysis | | including patients with outcome prior to the time window |
|  | | Kaplan - Meier survival analysis | | including patients with outcome prior to the time window |
|  | | Number of instances analysis | | including patients with outcome prior to the time window excluding patients with zero outcomes counts are grouped by date |
| Hyperkalemia | | | | |
|  | **Outcome definition** | | | |
|  | | Diagnosis | UMLS:ICD10CM:E87.5 | Hyperkalemia |
|  | **Settings for the performed analyses** | | | |
|  | | Kaplan - Meier survival analysis | | including patients with outcome prior to the time window |
|  | | Risk analysis | | including patients with outcome prior to the time window |
| Metabolic acidosis | | | | |
|  | **Outcome definition** | | | |
|  | | Diagnosis | UMLS:ICD10CM:E87.2 | Acidosis |
|  | **Settings for the performed analyses** | | | |
|  | | Kaplan - Meier survival analysis | | including patients with outcome prior to the time window |
|  | | Risk analysis | | including patients with outcome prior to the time window |
| Urine Retention | | | | |
|  | **Outcome definition** | | | |
|  | | Diagnosis | UMLS:ICD10CM:R33 | Retention of urine |
|  | **Settings for the performed analyses** | | | |
|  | | Risk analysis | | including patients with outcome prior to the time window |
|  | | Kaplan - Meier survival analysis | | including patients with outcome prior to the time window |
| eGFR (MDRD) | | | | |
|  | **Outcome definition** | | | |
|  | | Laboratory | TNX:8001 | Glomerular filtration rate/1.73 sq M.predicted [Volume Rate/Area] in Serum, Plasma or Blood by Creatinine-based formula (MDRD) ( (most recent occurrence)) |
|  | **Settings for the performed analyses** | | | |
|  | | Risk analysis | | including patients with outcome prior to the time window |
|  | | Lab values distribution | | patient's most recent lab result in the selected time window |
| eGFR- EPI | | | | |
|  | **Outcome definition** | | | |
|  | | Laboratory | UMLS:LNC:98980-6 | Glomerular filtration rate [Volume Rate/Area] in Serum, Plasma or Blood by Creatinine and Cystatin C-based formula (CKD-EPI 2021)/1.73 sq M ( (most recent occurrence)) |
|  | **Settings for the performed analyses** | | | |
|  | | Risk analysis | | including patients with outcome prior to the time window |
|  | | Lab values distribution | | patient's most recent lab result in the selected time window |
| Creatinine | | | | |
|  | **Outcome definition** | | | |
|  | | Laboratory | TNX:9024 | Creatinine [Mass/volume] in Serum, Plasma or Blood ( (most recent occurrence)) |
|  | **Settings for the performed analyses** | | | |
|  | | Risk analysis | | including patients with outcome prior to the time window |
|  | | Lab values distribution | | patient's most recent lab result in the selected time window |

### Definition of the “New-Onset” Analytic Frame

Each clinical outcome was analyzed under two pre-specified analytic frames, implemented through the TriNetX Compare Outcomes analytic. In the all-events frame the platform setting “including patients with outcome prior to the time window” was applied: all matched patients remain in the denominator regardless of whether the outcome was documented before the index date, and the resulting estimate reflects the total clinical burden of the outcome including recurrent events. In the new-onset frame the setting “excluding patients with outcome prior to the time window” was applied: any patient with any documentation of that specific outcome at any time before the start of the outcome window is removed from the denominator for that outcome.

Removal is outcome-specific. A patient excluded from the new-onset acute kidney injury analysis for having a prior N17 code remains eligible for the new-onset hyperkalemia analysis if no prior E87.5 code exists. The new-onset frame therefore isolates truly incident postoperative events and is the primary analytic frame for causal inference, because it eliminates prevalent-event contamination. Because patients are removed from the denominator, new-onset denominators are smaller than the matched cohort size and differ between outcomes and between arms. The observed denominators were 1,348 (robotic) versus 1,432 (laparoscopic) for urinary retention at 1 month, and at 6 months 1,016 versus 1,044 for acute kidney injury, 1,325 versus 1,329 for hyperkalemia, 1,418 versus 1,414 for metabolic acidosis, and 1,329 versus 1,415 for urinary retention.

TriNetX suppresses any cell containing fewer than 10 patients as a patient-privacy safeguard. At the 30-day horizon, once patients with a documented pre-index event were removed, the residual incident-event counts for acute kidney injury, MACE, metabolic acidosis, and hyperkalemia fell below this 10-event threshold in at least one arm and were therefore not released by the platform. Urinary retention was the only outcome whose incident event counts at 30 days exceeded the threshold in both arms and could be reported. Over the longer 180-day accrual window, incident-event counts for acute kidney injury, hyperkalemia, metabolic acidosis, and urinary retention all exceeded the suppression threshold in both arms, and the complete new-onset results are reported in Table 2 of the main manuscript. This is a platform-level data-release constraint rather than an analytic choice by the investigators.

### Secondary 6-Month Cohort

The 6-month analysis applied an identical cohort specification and an identical 61-covariate propensity score model, differing only in the restricted index window described above. Before matching the 6-month cohorts comprised 1,663 robotic and 7,152 laparoscopic patients; after 1:1 propensity score matching each arm comprised 1,659 patients. The index event and outcome definitions were unchanged from those specified above.
