# Supplementary material for: Robotic vs. laparoscopic inguinal hernia repair in patients with chronic kidney disease: a propensity score-matched analysis
Source: J Robot Surg. 2026 Aug 18;20(1):850. doi: 10.1007/s11701-026-03782-y (PMC13486028; doi:10.1007/s11701-026-03782-y)
Supplement: Supplementary file 3 [file 11701_2026_3782_MOESM3_ESM.docx]

# Table S.1. Subgroup Analysis of All Events (1-Month Follow-up)

| **Subgroup** | **N (per arm)** | **Outcome** | **Robotic Events** | **Lap Events** | **HR** | **95% CI** | **p-value** | **E-value (CI)** |
| --- | --- | --- | --- | --- | --- | --- | --- | --- |
| **Age ≥ 65** | 1,158 | MACE | 31 | 20 | 1.56 | (0.889, 2.736) | 0.121 | 2.49 (1) |
|  |  | AKI | 57 | 30 | 1.921 | (1.235, 2.990) | 0.003 | 3.25 (1.77) |
|  |  | Hyperkalemia | 19 | 15 | 1.271 | (0.646, 2.502) | 0.487 | 1.86 (1) |
|  |  | Met. Acidosis | 22 | 12 | 1.842 | (0.912, 3.722) | 0.088 | 3.09 (1) |
|  |  | Urine Retention | 54 | 47 | 1.142 | (0.773, 1.689) | 0.505 | 1.54 (1) |
| **Academic** | 1,336 | MACE | 33 | 11 | 3.027 | (1.530, 5.988) | 0.001 | 5.5 (2.43) |
|  |  | AKI | 68 | 42 | 1.647 | (1.121, 2.419) | 0.010 | 2.68 (1.49) |
|  |  | Hyperkalemia | 27 | 17 | 1.599 | (0.872, 2.934) | 0.129 | 2.58 (1) |
|  |  | Met. Acidosis | 28 | 16 | 1.763 | (0.954, 3.259) | 0.070 | 2.92 (1) |
|  |  | Urine Retention | 65 | 41 | 1.599 | (1.082, 2.364) | 0.018 | 2.58 (1.38) |
| **Obese (BMI ≥ 30)** | 805 | AKI | 38 | 22 | 1.759 | (1.041, 2.974) | 0.034 | 2.91 (1.25) |
|  |  | Hyperkalemia | 15 | 14 | 1.079 | (0.521, 2.235) | 0.837 | 1.37 (1) |
|  |  | Met. Acidosis | 17 | 12 | 1.432 | (0.684, 2.998) | 0.340 | 2.22 (1) |
|  |  | Urine Retention | 31 | 20 | 1.565 | (0.892, 2.745) | 0.118 | 2.51 (1) |
| **CKD Stage 1–3** | 1,092 | MACE | 22 | 21 | 1.051 | (0.578, 1.912) | 0.870 | 1.28 (1) |
|  |  | AKI | 40 | 28 | 1.437 | (0.887, 2.330) | 0.141 | 2.23 (1) |
|  |  | Urine Retention | 51 | 37 | 1.383 | (0.906, 2.112) | 0.133 | 2.11 (1) |
| **Dialysis Excl.** | 1,647 | MACE | 38 | 16 | 2.397 | (1.337, 4.299) | 0.003 | 4.23 (2.01) |
|  |  | AKI | 72 | 34 | 2.146 | (1.427, 3.226) | 0.0002 | 3.71 (2.21) |
|  |  | Hyperkalemia | 18 | 15 | 1.204 | (0.607, 2.390) | 0.595 | 1.7 (1) |
|  |  | Met. Acidosis | 23 | 13 | 1.779 | (0.901, 3.512) | 0.097 | 2.96 (1) |
|  |  | Urine Retention | 74 | 38 | 1.968 | (1.331, 2.909) | 0.0006 | 3.35 (1.99) |
| **Era 2020–2026** | 1,666 | MACE | 38 | 23 | 1.658 | (0.988, 2.782) | 0.055 | 2.7 (1) |
|  |  | AKI | 81 | 45 | 1.821 | (1.265, 2.622) | 0.0012 | 3.04 (1.84) |
|  |  | Hyperkalemia | 28 | 27 | 1.037 | (0.611, 1.760) | 0.892 | 1.23 (1) |
|  |  | Met. Acidosis | 29 | 18 | 1.616 | (0.897, 2.909) | 0.109 | 2.61 (1) |
|  |  | Urine Retention | 72 | 49 | 1.477 | (1.027, 2.123) | 0.035 | 2.32 (1.19) |
| **Initial Hernia** | 1,667 | MACE | 40 | 20 | 2.014 | (1.177, 3.444) | 0.01 | 3.44 (1.63) |
|  |  | AKI | 87 | 50 | 1.764 | (1.246, 2.497) | 0.001 | 2.92 (1.8) |
|  |  | Hyperkalemia | 33 | 26 | 1.277 | (0.764, 2.134) | 0.350 | 1.87 (1) |
|  |  | Met. Acidosis | 33 | 21 | 1.58 | (0.914, 2.731) | 0.101 | 2.54 (1) |
|  |  | Urine Retention | 75 | 43 | 1.758 | (1.208, 2.557) | 0.003 | 2.91 (1.71) |

Pre-specified subgroup analyses of clinical outcomes at 1-month follow-up, each subgroup independently propensity score-matched on the full set of 61 covariates. All events include patients with the outcome documented prior to the time window. Hazard ratios (HR) with 95% confidence intervals (CI) are from Cox proportional-hazards regression. E-values are reported for statistically significant associations; a protective effect estimate (HR <1) yields an undefined CI E-value.

Abbreviations: AKI, acute kidney injury; BMI, body mass index; CI, confidence interval; CKD, chronic kidney disease; Excl., excluded; HR, hazard ratio; MACE, major adverse cardiovascular events; Met., metabolic.

# Table S.2. Subgroup Laboratory Outcomes Post-operative Values (1-Month Follow-up)

| **Subgroup** | **Lab Marker** | **Robotic N** | **Robotic Mean ± SD** | **Lap N** | **Lap Mean ± SD** | **t** | **df** | **p-value** |
| --- | --- | --- | --- | --- | --- | --- | --- | --- |
| **Age ≥ 65** | eGFR MDRD | 366 | 52.451 ± 24.042 | 297 | 51.111 ± 23.066 | 0.727 | 661 | 0.467 |
|  | eGFR CKD-EPI | 21 | 53.962 ± 19.557 | 28 | 54.750 ± 21.230 | -0.133 | 47 | 0.895 |
|  | Creatinine | 366 | 1.807 ± 1.758 | 297 | 1.864 ± 1.734 | -0.419 | 661 | 0.675 |
| **Academic** | eGFR MDRD | 425 | 50.852 ± 24.852 | — | — | 0.499 | 806 | 0.618 |
|  | eGFR CKD-EPI | 26 | 55.931 ± 18.319 | — | — | 0.203 | 71 | 0.839 |
|  | Creatinine | 426 | 2.058 ± 2.166 | — | — | 0.079 | 812 | 0.937 |
| **Obese (BMI ≥ 30)** | eGFR MDRD | 280 | 51.720 ± 25.246 | 232 | 48.220 ± 25.353 | 1.558 | 510 | 0.120 |
|  | eGFR CKD-EPI | 18 | 58.935 ± 18.183 | 18 | 54.871 ± 19.696 | 0.643 | 34 | 0.524 |
|  | Creatinine | 280 | 1.933 ± 1.759 | 231 | 3.033 ± 11.616 | -1.563 | 509 | 0.119 |
| **CKD Stage 1–3** | eGFR MDRD | 325 | 57.376 ± 18.742 | 264 | 56.519 ± 16.728 | 0.579 | 587 | 0.563 |
|  | eGFR CKD-EPI | 16 | 60.892 ± 11.642 | 26 | 60.326 ± 16.073 | 0.122 | 40 | 0.903 |
|  | Creatinine | 325 | 1.356 ± 0.689 | 267 | 1.345 ± 0.619 | 0.198 | 590 | 0.843 |
| **CKD Stage 4–6** | eGFR MDRD | 43 | 31.135 ± 35.498 | 37 | 22.017 ± 21.704 | 1.358 | 78 | 0.178 |
|  | Creatinine | 44 | 4.883 ± 4.255 | 39 | 5.140 ± 3.836 | -0.288 | 81 | 0.774 |
| **Dialysis Excl.** | eGFR MDRD | 501 | 57.079 ± 22.144 | 382 | 55.666 ± 21.847 | 0.945 | 881 | 0.345 |
|  | eGFR CKD-EPI | 23 | 59.881 ± 17.953 | 30 | 59.228 ± 18.395 | 0.129 | 51 | 0.897 |
|  | Creatinine | 502 | 1.452 ± 0.847 | 385 | 1.996 ± 8.903 | -1.361 | 885 | 0.174 |
| **Recurrence** | eGFR MDRD | 83 | 54.726 ± 24.437 | 69 | 53.289 ± 24.539 | 0.360 | 150 | 0.719 |
|  | Creatinine | 83 | 1.831 ± 1.851 | 69 | 1.833 ± 1.756 | -0.006 | 150 | 0.996 |
| **Era 2015–2019** | eGFR MDRD | 52 | 44.991 ± 25.894 | 49 | 48.765 ± 28.500 | -0.697 | 99 | 0.487 |
|  | eGFR CKD-EPI | N\A | N\A | N\A | N\A | N\A | N\A | N\A |
|  | Creatinine | 52 | 2.583 ± 2.971 | 51 | 2.434 ± 2.451 | 0.278 | 101 | 0.782 |
| **Era 2020–2026** | eGFR MDRD | 527 | 53.632 ± 24.759 | N\A | N\A | 1.162 | 954 | 0.246 |
|  | eGFR CKD-EPI | 28 | 60.161 ± 24.128 | N\A | N\A | 1.515 | 62 | 0.135 |
|  | Creatinine | 529 | 1.867 ± 1.919 | N\A | N\A | -1.301 | 961 | 0.193 |
| **Initial Hernia** | eGFR MDRD | 522 | 52.034 ± 25.243 | N\A | N\A | 1.718 | 955 | 0.086 |
|  | eGFR CKD-EPI | 27 | 59.760 ± 24.492 | N\A | N\A | 0.895 | 58 | 0.374 |
|  | Creatinine | 524 | 1.977 ± 2.073 | N\A | N\A | -2.559 | 962 | **0.011** |

Post-operative laboratory outcomes stratified by pre-specified subgroups at 1-month follow-up. Values are reported as mean ± SD with N per arm; between-arm comparisons are by independent-sample t-test. Missing rows indicate subgroups with insufficient data for reliable estimation. Abbreviations: BMI, body mass index; CKD, chronic kidney disease; CKD-EPI, Chronic Kidney Disease Epidemiology Collaboration; df, degrees of freedom; eGFR, estimated glomerular filtration rate; Excl., excluded; MDRD, Modification of Diet in Renal Disease; SD, standard deviation

**Table S3 -Missing Labs.**

***Availability of Baseline Laboratory Covariates in the Propensity Score Model Before and After 1:1 Propensity Score Matching (Primary 1-Month Cohort).***

| **Laboratory Covariate** | **Code (LOINC/TNX)** | **Before Matching – Robotic (N=1,708)** | **Before Matching – Laparoscopic (N=6,853)** | **After Matching – Robotic (N=1,693)** | **After Matching – Laparoscopic (N=1,693)** |
| --- | --- | --- | --- | --- | --- |
| Body mass index (BMI) | TNX:9083 | 1,529 (89.5%) | 5,740 (83.8%) | 1,514 (89.4%) | 1,522 (89.9%) |
| Body weight | TNX:9081 | 1,357 (79.4%) | 5,363 (78.3%) | 1,345 (79.4%) | 1,349 (79.7%) |
| Heart rate | TNX:9074 | 1,478 (86.5%) | 5,371 (78.4%) | 1,463 (86.4%) | 1,451 (85.7%) |
| Oxygen saturation | TNX:9075 | 740 (43.3%) | 1,519 (22.2%) | 725 (42.8%) | 743 (43.9%) |
| Serum creatinine | TNX:9024 | 1,411 (82.6%) | 5,614 (81.9%) | 1,396 (82.5%) | 1,396 (82.5%) |
| eGFR (MDRD) | TNX:8001 | 1,409 (82.5%) | 5,555 (81.1%) | 1,394 (82.3%) | 1,399 (82.6%) |
| eGFR (CKD-EPI 2021) | LNC:98980-6 | 110 (6.4%) | 185 (2.7%) | 107 (6.3%) | 105 (6.2%) |
| Serum albumin | TNX:9045 | 1,145 (67.0%) | 4,358 (63.6%) | 1,132 (66.9%) | 1,151 (68.0%) |
| Platelet mean volume (MPV) | TNX:9019 | 1,075 (62.9%) | 3,528 (51.5%) | 1,061 (62.7%) | 1,045 (61.7%) |
| Prothrombin time (PT) | TNX:9033 | 352 (20.6%) | 1,274 (18.6%) | 343 (20.3%) | 354 (20.9%) |
| Hemoglobin A1c | TNX:9037 | 574 (33.6%) | 2,191 (32.0%) | 565 (33.4%) | 578 (34.1%) |
| LDL cholesterol | TNX:9002 | 597 (35.0%) | 2,416 (35.3%) | 588 (34.7%) | 586 (34.6%) |
| Troponin I (cardiac) | TNX:9005 | 24 (1.4%) | 192 (2.8%) | 24 (1.4%) | 28 (1.7%) |
| B-type natriuretic peptide (BNP) | TNX:9003 | 94 (5.5%) | 295 (4.3%) | 86 (5.1%) | 78 (4.6%) |
| NT-proBNP | TNX:9072 | 91 (5.3%) | 246 (3.6%) | 88 (5.2%) | 80 (4.7%) |
| Corrected QT interval (QTc) | TNX:2001 | 387 (22.7%) | 1,225 (17.9%) | 382 (22.6%) | 378 (22.3%) |
| Urine albumin/creatinine ratio | LNC:LG34557-5 | 54 (3.2%) | 140 (2.0%) | 51 (3.0%) | 52 (3.1%) |

Values are the number of patients (n) in each cohort with at least one recorded value for the specified laboratory covariate within the pre-specified baseline lookback window (proportion of the cohort in parentheses). Availability was extracted directly from the TriNetX Global Collaborative Network cohort characteristics table for the primary 1-month cohort (query executed July 20, 2026).

Handling of missing values: In accordance with the TriNetX propensity score workflow, laboratory covariates with missing values were handled using a complete-case within-covariate approach. Patients without a recorded value for a given laboratory covariate within the pre-specified baseline lookback window were excluded from the matching distance calculation for that covariate only; they remained eligible for matching on all other covariates for which values were available. No imputation was performed. Consequently, balance on each laboratory covariate is achieved only among patients with an available value, and reduced denominators for several laboratory measures (e.g., eGFR CKD-EPI 2021, urine albumin/creatinine ratio, troponin I, BNP, NT-proBNP) contribute to the residual post-matching standardized mean differences reported in Table 1 and in the manuscript Limitations.

Abbreviations: BMI, body mass index; BNP, B-type natriuretic peptide; CKD-EPI, Chronic Kidney Disease Epidemiology Collaboration; eGFR, estimated glomerular filtration rate; LDL, low-density lipoprotein; LNC, LOINC; MDRD, Modification of Diet in Renal Disease; MPV, mean platelet volume; NT-proBNP, N-terminal pro–B-type natriuretic peptide; PT, prothrombin time; QTc, corrected QT interval; TNX, TriNetX platform code.

# Supplementary Figure S1. Decomposition of Kaplan-Meier Cumulative Incidence into True CIF and Bias from Censoring Death.

Figure S1 legend. Stacked-bar decomposition of the Kaplan-Meier (KM) cumulative-incidence estimate at 6 months for each of the five pre-specified outcomes, separately for laparoscopic and robotic-assisted cohorts (N = 1,659 per arm). The blue segment represents the true competing-risk cumulative incidence function (CIF), which explicitly treats all-cause death within the follow-up window as a competing event. The red segment represents the additional cumulative-incidence overestimate produced by the Kaplan-Meier estimator, which treats death as non-informative censoring. Across all five outcomes, the KM–CIF discrepancy is approximately 1.1–1.9 percentage points, driven by the low observed mortality rates in the two arms (Robotic: 25/1,659 [1.5%]; Laparoscopic: 31/1,659 [1.9%]). The direction and rank order of every outcome are preserved when moving from KM to competing-risk CIF, indicating that the primary results reported in the main manuscript are robust to competing-risk adjustment. CIF was approximated from aggregate event and death counts because TriNetX releases only de-identified summary statistics, precluding individual-level Fine-Gray subdistribution hazard modeling.
